# Effectiveness of rosemary extract on the cardiovascular risk of emergency nursing professionals – an intervention study^*^

**DOI:** 10.1590/1980-220X-REEUSP-2025-0167en

**Published:** 2026-03-16

**Authors:** Carolina Renz Pretto, Rosângela Marion da Silva, Juliana Tamiozzo, Luciane Flores Jacobi, Matias Nunes Frizzo, Christiane de Fátima Colet, Flávia Camef Dorneles Lenz, Silviamar Camponogara

**Affiliations:** 1 Secretaria Municipal de Saúde de Ijuí, Ijuí, RS, Brazil.; 2 Universidade Federal de Santa Maria, Santa Maria, RS, Brazil.; 3 Universidade Regional do Noroeste do Estado do Rio Grande do Sul, Ijuí, RS, Brazil.

**Keywords:** Rosmarinus, Complementary Therapies, Cardiovascular Diseases, Heart Disease Risk Factors, Emergency Nursing

## Abstract

**Objective:**

To analyze the effectiveness of the oral administration of rosemary dry extract capsules (*Rosmarinus officinalis L*.) (1g/day) on the estimated cardiovascular risk, over ten years, in nursing professionals working in emergency and urgent care services.

**Method:**

Quasiexperimental study conducted in Southern Brazil. Participants completed a sociodemographic and clinical questionnaire, had blood collected for glycemic and lipid profile analysis, and took oral capsules containing 500 mg of dry rosemary extract twice a day for 8 weeks; subsequently, a new blood sample was taken. The Framingham Global Risk Score was used to estimate cardiovascular risk. Data analysis used descriptive and inferential statistics; significance level of 5%.

**Results:**

The study included 36 professionals, predominantly obese and those with elevated total cholesterol. When comparing the variables before and after the intervention, differences were found in blood pressure (p = 0.048), in total cholesterol (p < 0.001), and in the Framingham Global Risk Score (p = 0.047).

**Conclusion:**

Dry rosemary extract was effective in reducing the estimated cardiovascular risk of nursing professionals. Brazilian Registry of Clinical Trials No. RBR 88hrnnw.

## INTRODUCTION

Death from cardiovascular disease serves as a warning to healthcare systems. It accounts for 33% of all deaths, mainly due to myocardial infarction and stroke. The causes involve a set of socioeconomic, environmental, behavioral, and genetic factors, such as unhealthy diet, stress, sedentary lifestyle, high blood pressure, diabetes, obesity, sex, age, and others^(1)^. Cardiovascular risk can be determined using the Framingham Global Risk Score (FRS), which estimates the occurrence of coronary, cerebrovascular, peripheral arterial disease, or other events over 10 years^(2)^.

In Emergency Care Units (*UPA*), the nursing team’s work is characterized by providing rapid care to patients with acute or exacerbated chronic conditions, which can result in suffering, sequelae, or death^(3)^. This context demands skill and technical-scientific knowledge to carry out activities involving embracement, risk classification, stabilization, referral, management, education, and research^(3)^. These organizational characteristics and work processes make nursing professionals working in these services especially vulnerable to developing health problems. Therefore, this is a population at higher risk for these diseases, particularly due to healthcare pressure, intense physical and emotional demands, and a fast pace of work. A study identified that 52.9% of nursing professionals in an *UPA* unit were overweight or obese, 61.8% had increased abdominal circumference, 58.8% were sedentary, 79.4% reported moderate to very high stress levels, and 82.3% had poor sleep quality^(4)^. This evidence reinforces the importance of investigating health conditions in this group.

This caregiving scenario presents inadequate working conditions, harmful relationships, lack of material and human resources, professional devaluation, and psychological distress^(5)^; shift work organization, employees with poor sleep quality, and altered cortisol levels^(6)^. Considering these aspects, research has shown a relationship between shift work and increased blood pressure, total cholesterol, and body mass index, and has identified shift work as a risk factor for coronary artery disease^(7)^. Another study showed an association between elevated levels of hair cortisol and acute myocardial infarction^(8)^.

The pursuit of well-being has required therapeutic approaches that complement conventional ones, including the use of medicinal plants. This use is an ancient practice, but the recognition of medicinal plants by the World Health Organization (WHO) as a therapeutic resource dates back to 1978. Since then, WHO has consistently encouraged the formulation of policies for their use in healthcare^(9)^, which has required further research. In Brazil, the National Policy on Integrative and Complementary Practices in the Brazilian Public Health System (*PNPIC*), implemented in 2006 and updated in 2018^(10)^, and the National Policy on Medicinal Plants and Herbal Medicines (*PNPMF*), established in 2006, has expanded the use of and access to these therapies^(11)^. Rosemary (*Rosmarinus officinalis L*.) is an aromatic plant, multifaceted in nature and with versatile applications, consisting of various active compounds, phenolic diterpenes, flavonoids and essential oil, with anti-inflammatory and antioxidant properties^(12)^ that deserve to be explored more thoroughly as a therapeutic tool.

The literature indicates a limited number of studies that have evaluated the effect of rosemary on cardiovascular risk factors. Research with patients with diabetes mellitus showed that the ingestion of rosemary tea reduced waist and hip measurements, the percentage of glycated hemoglobin, and insulin resistance among the participants^(13)^. Another study, involving healthy individuals and those undergoing treatment for diabetes mellitus, showed a reduction in blood glucose levels and an increase in vitamin B12 concentration in both groups^(14)^. In healthy individuals, research has shown a decrease in blood glucose levels with the ingestion of rosemary powder and a reduction in blood lipid levels^(15)^.

Considering the work context of emergency nursing professionals and the limited evidence available on rosemary and its therapeutic potential, and the need for safe products to reduce cardiovascular risk and morbidity and mortality from cardiovascular diseases, the objective of this study was to analyze the effectiveness of oral administration of dry rosemary (*Rosmarinus officinalis L*.) extract capsules (1g/day) on the estimated cardiovascular risk, over ten years, in nursing professionals working in emergency and urgent care services.

## METHOD

### Study Design and Local

This is a quasi-experimental intervention study with a single pre- and post-test group, which followed the recommendations of *Consolidated Standards of Reporting Trials* (CONSORT). The study involved nursing professionals working in emergency services in an inland city of the state of Rio Grande do Sul, Brazil, namely, an Urgent Care Unit and a Hospital Emergency Room, totaling 100 professionals, including nurses and nursing technicians, under any form of employment contract. The *UPA* attends to all types of needs and performs approximately 4,800 consultations per month; the hospital emergency service is a health reference in the region where it is located, considered a high-complexity care center, and serves a population of approximately 1.5 million people.

### Participants

Among those interested in participating in the study, the following were excluded: those with hypersensitivity to rosemary or components of the herbal formulation, pregnant women, breastfeeding women, epileptics, those undergoing treatment for dyslipidemia, those diagnosed with diabetes mellitus, biliary or hepatic dysfunction, prostatic diseases or gastroenteritis, and those absent from work for any reason during the data collection period. The sampling criteria were established considering the recommendations of the Brazilian Health Regulatory Agency regarding the use of rosemary^(16)^ and to avoid potential confusion between the results of treatments for pre-existing conditions and the effects of rosemary on the outcomes analyzed.

### Sample Size

For sample size determination, a 2-point difference between FRS values before and after the intervention, an 80% chance of type II error (test power) 5% significance, and a standard deviation of 3.2 were considered, as per a study with nurses from a university hospital^(17)^. Due to potential losses, 20% were added to the minimum sample size. Therefore, the established sample size was 50 workers.

### Data Collection

Data collection took place from August to November 2022 and was carried out at the participants’ workplace, in a private room. Sociodemographic data were collected: age, biological sex, self-reported skin color, family income, marital status, education level, job title, work shift, and work hours; clinical variables: abdominal circumference (in centimeters), blood pressure (in mmHg), and smoking status.

Abdominal circumference (AC) was measured with the participant standing upright, hands at their sides, using a body circumference measuring tape with a capacity of 1.5 m/0.1 cm, positioned at the level of the navel with the abdomen relaxed, after exhalation. The parameters used as a reference for AC were those characterizing central obesity in the South American population, as measured by > 90 cm for men and > 84 cm for women.

Blood pressure (BP) checks were performed manually, with the participant seated, using a calibrated aneroid sphygmomanometer positioned on the right upper limb supported by a cuff, brachial perimeter elevated at heart level, legs uncrossed, feet flat on the floor, empty bladder, and stethoscope. Two blood pressure measurements were taken on the same arm, the first at the start of the work shift and the second one minute after the first.

Based on the measurements, the average Systolic Blood Pressure (SBP) and Diastolic Blood Pressure (DBP) were calculated to get as close as possible to the participants’ actual blood pressure values. The BP was classified according to the Update of the Cardiovascular Prevention Guideline of the Brazilian Society of Cardiology^(2)^, in which SBP values above 120 mmHg and DBP values above 80 mmHg are considered high.

Blood samples were collected to measure total cholesterol (TC), high-density lipoprotein cholesterol (HDL-c), and glycated hemoglobin (HbA1c) levels. Desirable fasting total cholesterol levels were considered to be less than 190 mg/dL and HDL-c levels greater than 40 mg/dL. HbA1c values below 5.7% were classified as normal, and values ≥ 5.7% as altered.

The day before the blood collection, participants were instructed via email, through an app, to maintain a 12-hour fast beforehand. From the brachial vein, 5 mL of blood were collected by a trained professional from the contracted laboratory, transferred to a vacutainer tube without anticoagulant for analysis of biochemical parameters, and submitted to Cobas Integra 400 automation using Roche supplies.

Cardiovascular risk stratification was performed using the FRS for men and women, which estimates the occurrence of cardiovascular events over 10 years^(2)^. This method considers data on sex, age, HDL-c concentration, TC, smoking, diabetes mellitus (DM), and blood pressure. Each data point receives a score based on the values presented by each individual, which, when added together, allow for the establishment of the cardiovascular risk percentage. Individuals with FRS <10% are considered to be at low risk, 10%–20% at medium risk, and >20% at high risk.

FRS starts scoring age from 30 years old, meaning the 30–34 age group receives a score of zero. Given the limited population size of this study and to avoid excluding participants, all those under 30 years of age also received a score of zero.

### Intervention

The intervention took place after the first blood sample was taken and the other data collection instruments were completed. All participants received two bottles containing a total of 56 capsules with 500 mg of dry rosemary extract each, to be used twice a day (total dose of 1 g/day), for a period of eight weeks. It was recommended to take the first capsule in the morning, 30 minutes after breakfast, and the second, 12 hours after the first, with water. For participants who did not habitually eat breakfast, it was recommended that capsule administration occur 30 minutes post-awakening.

The capsules were produced by a Teaching Pharmacy contracted for this purpose. For the production of the capsules, the *Rosmarinus officinalis L*. dry extract PS 0.03% was used, composed mainly of pinene, camphene, cineole, borneol, bornyl acetate, camphor, diterpenes, saponin, traces of alkaloids, bitter ingredients and tannins. The *Rosmarinus officinalis L*. dry extract P.S. 0.03% originates from the leaves of the plant, with the appearance of a fine hygroscopic powder without the presence of foreign materials, greenish-beige in color, with a characteristic odor and flavor, with total ash in values lower than 5.0%, density between 0.300 and 0.600, pH between 4.0 and 8.0, partially water-soluble, with humidity lower than 10.0%, active flavonoid content greater than 0.03, aerobic bacteria count lower than 10,000, fungi or yeast count lower than 1,000, and enterobacteria count lower than 1,000. The capsules were made with odorless and tasteless gelatin, filled with 500 mg of dry rosemary extract and starch (to complete filling, if necessary).

For greater safety and control regarding adherence to the proposed treatment and identification of adverse events, each participant was provided with a mini-diary to record the time of self-administered, forgotten or late doses, and possible reactions, as well as an infographic with guidelines on: maintaining the use of routine medications, dietary habits and physical activity; doses and times of self-administration of the herbal medicine; what to do in case of a forgotten dose or adverse reaction; storage precautions; and contact information for the researchers. All participants were instructed to keep any untaken capsules, in case they forgot, to return them to the researchers at the end of the intervention period, as a way to control the doses ingested.

During the intervention period, participants were contacted weekly via messaging app for follow-up, encouragement to use the herbal remedy, and maintenance of routine treatments.

Regarding losses to follow-up, participants who dropped out of the study before completing eight weeks of intervention use and those who, even after completing the dosage regimen, refused to undergo laboratory tests or did not complete the self-administered questionnaires were considered.

### Data Analysis and Treatment

For data analysis, FRS was considered the primary outcome, and BMI, AC, SBP, DBP, HbA1c, TC, LDL-c, and HDL-c were considered secondary outcomes. The collected data formed a bank in the software Microsoft Excel^®^ through independent double typing, and were analyzed with the help of the software SPSS, version 21.0 and the software Jamovi, version 2.3.26.

Qualitative variables were expressed as absolute and relative frequencies; quantitative variables were presented as mean and standard deviation in the presence of a normal distribution, or median and interquartile range in the presence of an asymmetrical distribution. Data normality was verified using the Shapiro-Wilk test. The comparison of quantitative variables before and after the intervention was performed using the Student’s t-test for paired samples, considering a normal distribution, or the Wilcoxon test, in the case of an asymmetrical distribution. P values <0.05 were considered statistically significant.

As measures of effect size, Cohen’s d (d) was used together with the Student’s t-test for paired samples or biserial rank correlation, combined with the Wilcoxon test. Classified as a small effect, Cohen’s d values equal to 0.2; medium when d = 0.5; and large, d = 0.8. For the biserial correlation, small, medium, and large effects were considered to be 0.10, 0.24, and 0.37, respectively^(18)^.

Mixed-effects analysis of variance (mixed ANOVA) was used to compare differences between paired groups from unpaired categories. P values <0.05 were considered statistically significant. The effect size was measured using the generalized Eta-squared (n2G) test, where values of 0.01 are classified as small, 0.06 as medium, and 0.140 as large^(18)^.

### Ethical Aspects

All the assumptions of the 1984 Declaration of Helsinki and Resolution No. 466/2012, which govern research with people in Brazil, were followed. The research project was approved by the Research Ethics Committee of the Universidade Federal de Santa Maria under opinion number 5.197.916 and was registered in the Brazilian Registry of Clinical Trials – Rebec, no. RBR 88hrnnw. Free and informed consent was obtained from participants in the first and second stages of data collection.

## RESULTS

**Figure 1:**
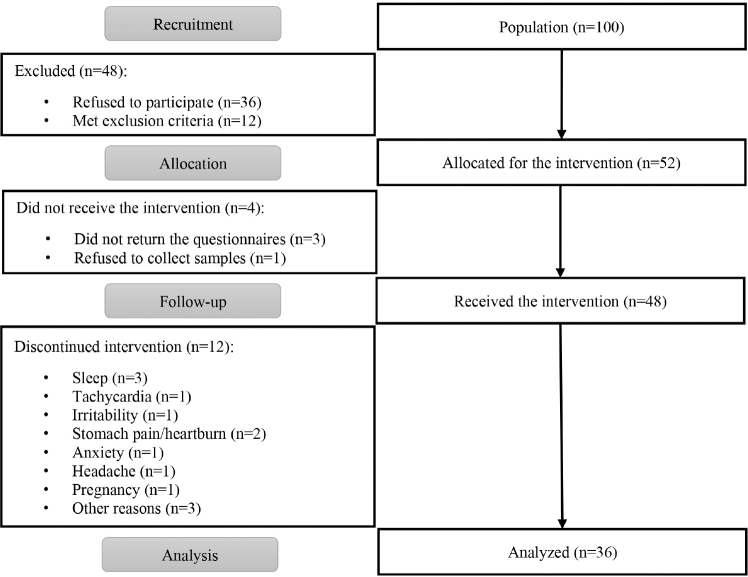
Flowchart for the composition of the sample of nursing professionals who participated in the quasi-experimental study.

Source: Adapted from the CONSORT 2010 Flow Diagram.

Of the population invited to participate in the study (n = 100), 36 refused to participate and 12 did not meet the selection criteria. After data collection began with 52 participants, three did not return the self-administered questionnaires and one refused to have blood drawn, therefore being excluded (Figure 1).

The intervention began with 48 participants. After four weeks of intervention, 12 professionals discontinued treatment and did not participate in the final data collection phase. The final sample consisted of 36 participants. The professionals justified the discontinuation of the herbal medicine due to drowsiness, tachycardia, anxiety, irritability, headache, stomach pain/heartburn, personal reasons, and pregnancy. One participant reported improvement in facial skin and continued therapy until the end.

Regarding the characteristics of the professionals, the predominant sex was female, and the age < 40 years old, white skin color, and having a partner (Table 1). Regarding job characteristics, most were employees with a high-school level job, but with higher education, working rotating shifts and with working hours > 44 hours per week.

**Table 1:** Socio-labor characteristics of nursing professionals in emergency services – Ijuí, RS, Brazil 2022 (n = 36).

Socio-labor variables	Frequency	
	N	%
**Biological sex**		
Male	6	16.7
Female	30	83.3
**Age**		
≤ 40 years	22	61.1
> 40 years	14	38.9
**Self-reported skin color**		
White	30	83.3
Other	6	16.7
**Marital status**		
With a partner	26	72.2
Without a partner	10	27.8
**Monthly family income**		
≤ 5,038 reais (≤ US$ 1,618.68)	13	36.1
> 5,038 reais (> (US$ 1618.68)	23	63.9
**Level of education**		
High school	11	30.6
Undergraduate or graduate degree	25	69.4
**Position**		
Nursing assistants and technicians	28	77.8
Nurse	8	22.2
**Work shift**		
Fixed	9	25.0
Rotating	27	75.0
**Workload**		
≤ 44 hours per week	6	16.7
> 44 hours per week	30	83.3
**Total**	36	100.0

Regarding clinical variables, obese nursing professionals predominated, with normal blood pressure, elevated total cholesterol, and desirable HDL-c levels (Table 2).

**Table 2:** Clinical variables and laboratory parameters of nursing professionals working in emergency services – Ijuí, RS, Brazil, 2022 (n = 36).

Clinical variables and laboratory parameters	Frequency	
	N	%
**Abdominal circumference**		
Normal	8	22.2
Central obesity	28	77.8
**Blood pressure**		
Normal	26	72.2
High	10	27.8
**Use of antihypertensive medication**		
Yes	4	11.1
No	32	88.9
**Smoking**		
Yes	4	11.1
No	32	88.9
**Glycated Hemoglobin**		
Normal	27	75.0
Changed	9	25.0
**Total cholesterol**		
Desirable	14	38.9
High	22	61.1
**High-Density Lipoprotein Cholesterol**		
Desirable	32	88.9
Reduced	4	11.1

When comparing the clinical variables and laboratory parameters of the participants before and after the intervention with rosemary, significant differences were found in the variables blood pressure and cholesterol (Table 3).

**Table 3:** Effectiveness of rosemary on clinical variables: comparison before and after the intervention – Ijuí, RS, Brazil, 2022 (n = 36).

Clinical variables and laboratory parameters	Intervention with rosemary		Effect size	p value
	Before Mean ± SD	After Mean ± SD
Abdominal Circumference (cm)	98.08 ± 16.80	97.38 ± 15.38	0.091^a^	0.637^**^
Systolic blood pressure (mmHg)	117.03 ± 11.10	113.28 ± 13.39	0.342^b^	0.048^*^
Diastolic blood pressure (mmHg)	78.31 ± 7.68	74.36 ± 9.10	0.423^b^	0.016^*^
Total cholesterol (mg/dL)^c^	200.62 ± 29.87	185.25 ± 25.40	0.649^a^	<0.001**
High-density lipoprotein cholesterol (mg/dl)	71.69 ± 24.11	57.41 ± 19.38	1.03^b^	<0.001^*^
Glycated hemoglobin (%)	5.43 ± 0.42	5.42 ± 0.33	0.054^b^	0.746^*^

Notes: *T test for paired samples;

**Wilcoxon test;

^a^ Biserial correlation of ranks;

^b^ D for Cohen;

^c^ n = 35.

A significant reduction in mean systolic and diastolic blood pressure was identified, with an effect size ranging from small to medium. It is noteworthy that PAS remained unchanged in 16.7% (n = 6), decreased in 50.0% (n = 18), and increased in 33.3% (n = 12). Diastolic blood pressure remained constant in 25.0% (n = 9), decreased in 55.6% (n = 20), and increased in 19.4% (n = 7) of the professionals.

TC parameters showed a significant decrease with a large effect; among nursing professionals, 74.3% (n = 26) showed a reduction after using rosemary, and 25.7% (n = 9) of them showed an increase. HDL-c levels decreased significantly, with a large effect size. There was a reduction in serum HDL-c levels in 86.1% (n = 31) of professionals, an increase in 11.1% (n = 4), and 2.8% (n = 1) maintained constant values.

In the FRS analysis, a global median of 3.5 points (IQR = 6.00) was identified; 7.5 (IQR = 11.5) for men and 2 (IQR = 7.0) for women, with a difference between the sexes (U = 34.50, p = 0.018). For analysis of the effect of rosemary on the FRS, the factors sex and age were considered (Table 4). It was found that professionals of the male biological sex have a higher risk for cardiovascular events in 10 years (p < 0.001), as well as those of age > 40 years (p < 0.001), with a large effect size.

**Table 4:** Effectiveness of rosemary on the Framingham Global Risk Score, comparison between intervention periods, according to sex and age – Ijuí, RS, Brazil, 2022 (n = 35).

	Period	Biological sex		p value^*^ sex effect (n^2^G)	p value^*^ intervention-sex interaction (n^2^G)	Age		p-value^*^ effect of age (n^2^G)	p value^*^ intervention-age interaction (n^2^G)	p value^*^ effect of the intervention (n^2^G)	p-value interaction intervention-sex-age (n^2^G)
		Male Mean ± SD	Female Mean ± SD			< 40 years Mean ± SD	> 40 years Mean ± SD				
**FRS**	Before	9.16 ± 6.85	2.31 ± 4.00	**<0.001** (0.382)	0.970 (0.00)	0.77 ± 2.99	8.07 ± 4.94	**<0.001** (0.474)	0.063 (0.015)	**0.047** (0.018)	0.440 (0.003)
	After	8.17 ± 5.19	1.45 ± 3.65			0.41 ± 3.06	6.31 ± 4.57				

Note: FRS – Framingham Global Risk Score; SD – Standard Deviation;

^*^Mixed-effects ANOVA and effect size - Generalized Eta squared (n^2^G).

The intervention with rosemary significantly reduced the FRS score (p = 0.047); however, the effect size was small (n2G = 0.018). It should be noted that 28.6% (n = 10) of professionals maintained the FRS unchanged between the observation periods, 51.4% (n = 18) showed a decrease, and 20.0% (n = 2) showed an increase.

## DISCUSSION

The main result of this study is that the ingestion of dry extract capsules of *Rosmarinus officinalis L*. (1g/day) may contribute to reducing the risk of cardiovascular events in emergency department nursing professionals, mainly by promoting a decrease in blood pressure and total cholesterol with few adverse effects, but the findings are preliminary and should be interpreted with caution. It is recognized that CVDs are the leading cause of morbidity and mortality in Brazil and worldwide, and dyslipidemia and hypertension stand out among their risk factors^(19)^; and effective herbal remedies in reducing these factors can produce positive impacts.

The results contribute to the consolidation of the nursing science; expand the evidence on the use of rosemary in the treatment of risk factors for atherosclerosis and cardiovascular diseases, which have been little explored until now; contribute to scientific development based on cultural diversity; validate popular knowledge about the benefits of rosemary; and encourage the leading role of nursing in research with herbal medicines.

The findings of this investigation were based on a sample in which the socio-labor characteristics regarding age and sex resemble the profile of nursing worldwide, which is predominantly female and relatively young^(20)^. Their educational level, with a prevalence of higher education or above, even though most professionals held high-school level positions, reflects changes in the nursing job market, which demands professionalization, specialization, and encourages competitiveness^(21)^.

The average monthly family income of approximately US$1,618.68 (± US$677.15) or R$5,838.00 allowed nursing to be classified as middle class^(22)^, which seems to be a good indicator; however, it was found that most professionals worked more than 44 hours a week and worked rotating shifts, putting their health at risk. In this regard, a study found an association between daytime work with a routine return to work in less than 11 hours and the risk of myocardial infarction (MI)^(23)^.

It was found that more than 50% of the professionals were overweight or obese, had increased abdominal circumference, altered total cholesterol levels, and almost 30% had alterations in systemic blood pressure. These risk factors may be related to the organization of nursing work. Research reinforces this assertion, indicating an association between shift work and increased blood pressure, total cholesterol, and BMI^(7)^. Similarly, dysregulation in cortisol secretion and chronic stress, common among these professionals, are linked to epigenetic changes involved in dyslipidemia, insulin resistance, adiposity, impaired immune response, and increased systemic inflammation^(24)^.

Despite this context, the participants presented a low risk for cardiovascular events over 10 years. A relationship was identified between the estimated cardiovascular risk and age and sex, with higher risk in men and those over 40 years of age. This finding supports current evidence that the occurrence of cardiovascular disease increases with population aging and that the global mortality rate from CVD among men is higher compared to women^(1)^.

Chronological age is a risk factor, but there is interaction and synergy between chronological age and modifiable risk factors in the development of CVD, reinforcing the importance of preventive measures and those that promote healthy aging. Regarding sex, it is understood that, although there is a lower prevalence of CVD in women than in men, after an acute cardiovascular event women have a higher mortality rate and a worse prognosis. Also noteworthy is the influence of the genetic expression of sex chromosomes and differences in sex hormones on the pathophysiology and occurrence of cardiovascular events^(25)^.

Regarding the effectiveness of rosemary dry extract on risk factors for cardiovascular disease (CVD), when comparing the period before and after the intervention, a significant reduction in systolic and diastolic blood pressure was observed in nursing professionals. Compounds in rosemary, such as rosmarinic acid, act to reduce angiotensin II and angiotensin-converting enzyme levels, which are related to increased blood pressure^(26)^, may have contributed to the reduction in systolic and diastolic blood pressure in the participants.

Considering the high prevalence of hypertension in the population and its causal relationship with ischemic heart disease and mortality from stroke, which result in years of life lost due to disability^(19)^, the hypotensive potential of rosemary may contribute to reducing these outcomes. However, the effect observed in the analyzed sample was small to medium in size, indicating limited clinical and practical importance.

In the present study, no statistically significant difference was identified between the intervention periods regarding abdominal circumference, although there was a reduction in their average values, which may be related to the dose used and/or the intervention time. A study with obese women involving the ingestion of rosemary oil capsules at a dose of 380 mg/day for six weeks, combined with diet, showed a reduction in the waist-to-hip ratio^(27)^, as also observed in people with DM who used rosemary tea at a dose of 2g/day for 12 weeks^(13)^. Considering that the waist-to-hip ratio is associated with excess weight, metabolic syndrome, and diabetes mellitus, reducing this measurement, linked to the use of Rosemary, may result in a reduction in cardiovascular risk.

Total cholesterol levels were also lower after the intervention, with a large effect size, suggesting clinical significance. A similar finding was verified in a study that evaluated the effect of rosemary powder at doses of 5 and 10 g/day for 8 weeks on the lipid profile of healthy individuals^(15)^. The mechanism of action of rosemary, involved in lowering cholesterol and triglycerides, is linked to its anti-inflammatory and antioxidant capacity, which interferes with lipid metabolism, inhibits lipolysis and adipocyte maturation, reduces the synthesis of cholesterol and fatty acids in the liver and adipose tissue, and increases their clearance^(28)^.

Based on the mechanism of action of rosemary, it can be inferred that the results of this research reinforce the evidence regarding the plant’s potential for vascular health, since the participation of cholesterol in atherosclerotic processes is related to the individual’s capacity to absorb this lipid, the type of absorption that contributes to the composition and quality of LDL-c particles, and its ineffective elimination^(29)^.

Regarding HDL-c, when comparing the intervention periods, a significant reduction in the parameter was observed, with a large effect. Research has suggested that, before menopause, elevated HDL-c levels are cardioprotective for women, but not for those transitioning to menopause. Reduced estradiol levels influence factors affecting HDL-c particle quality, composition, and function, making it a pro-inflammatory and pro-atherogenic factor, independent of age, race/ethnicity, education, BMI, systolic blood pressure, LDL-c, use of CVD medications, and C-reactive protein^(30)^.

Research conducted with Chinese adults revealed a U-shaped association between HDL-c and mortality from all causes, cardiovascular disease, and cancer. It has been shown that one in thirty deaths from all causes can be attributed to high and low levels of HDL-c, and that values between 50 and 79 mg/dL represent a lower risk of mortality from all causes and from CVD. High and low levels of HDL-c have been associated with increased mortality from ischemic heart disease and cerebrovascular ischemic disease. The authors also point to the inflammatory and atherogenic potential of the molecule when it undergoes alterations in protein composition and becomes dysfunctional^(31)^. Therefore, a beneficial effect of rosemary on this outcome is suggested, as the average HDL-c values of the participants remained within the normal range. However, the effect of rosemary on this molecule should be investigated in the long term, considering that the progressive reduction of HDL-c levels can be harmful to cardiovascular health and to support questions regarding the plant’s safety.

No significant changes in glycated hemoglobin were observed between the intervention periods, consistent with the study involving healthy individuals and those with diabetes^(14)^, in which the use of 3 g/day of powder of encapsulated *Rosmarinus officinalis L.* for 8 weeks in healthy individuals showed no significant difference with the intervention. Conversely, studies suggest that rosemary and its phenolic compounds improve insulin resistance, suppress gluconeogenesis, stimulate glucose uptake, and have anti-inflammatory and antioxidant effects^(32)^, highlighting the need for investigations with longer intervention times or with different doses than the one studied to clarify the effectiveness of rosemary on this parameter.

The intervention resulted in a significant reduction in the FRS score, however, with limited clinical-practical significance. It is noteworthy that 51.4% of professionals showed a decrease in their score, which may be associated with a reduction in the blood pressure and total cholesterol parameters identified in the participants, considering that the algorithm takes these variables into account to estimate risk^(2)^.

Worldwide, more than half a billion people are affected by cardiovascular diseases, primarily ischemic heart disease and stroke, and medical advances in this area are concentrated in high-income countries^(1)^. Research aimed at providing evidence for a complementary or adjuvant therapy in the treatment of risk factors for these diseases, easily accessible to the population in a developing country, is an innovation that contributes to reducing premature mortality from chronic non-communicable diseases, a target of the Sustainable Development Goals.

Regarding the intervention with rosemary, the analysis of its effectiveness on the health outcomes of nursing professionals, with a view to the safety of the participants, was carried out in conjunction with the monitoring and identification of adverse events. Events such as drowsiness, improved facial skin, tachycardia, anxiety, irritability, frequent crying, headache, and stomach pain/heartburn were possibly related to the intervention, unlike what was observed in a study with incarcerated individuals^(33)^, who, with a similar intervention, did not identify any adverse events. These findings reinforce the need for further research evaluating its safety and effectiveness in different outcomes. They highlight the importance of actions to promote its rational use and education on the correct use of botanical preparations, the adherence to dosage, the route of administration, duration of use and demystification of harmlessness.

Different botanical preparations, such as dry extracts and essential oils, can produce different effects on the body, as can the concentration of certain chemical compounds in these substances. An example of this was the result presented by research with rosemary directed at people diagnosed with primary hypotension, in which 1 ml of essential oil on a sugar cube every 8 hours for 44 weeks showed a tendency to increase blood pressure^(34)^, unlike the hypotensive effect found in this study with the dry extract containing an active flavonoid content greater than 0.03. It is important to underscore that the essential oil is an aromatic, volatile product, a secondary metabolite used by plants for protection, whose effectiveness depends on the concentration and synergistic interaction of its chemical constituents, influenced by extraction processes, the plant’s growing environment, and other factors^(35)^. Dry extract, in turn, is a solid preparation obtained from plant drugs, resulting from the evaporation of the solvent used in the extraction process and the elimination of undesirable materials, and has specifications of chemical constituents (for quality control purposes)^(16)^.

The execution of this research presented some limitations: the small sample size due to loss to follow-up, which may introduce attrition bias; the FRS having low sensitivity for predicting short-term events; the lack of systematic monitoring of relevant behavioral variables, such as diet, physical activity, concomitant use of medications, and stress levels or sleep quality, with potential confounding bias; and the absence of a control group and the evaluation of the effectiveness of rosemary on glycated hemoglobin with an intervention that lasted less than 3 months, which may have influenced the results. Furthermore, the small effect size observed on FRS and blood pressure indicates a limited clinical effect for these outcomes. Despite this, the results provided evidence with the potential to produce social and economic impact, as well as changes in morbidity and mortality indicators.

Given the limitations and the fact that this is a preliminary study, the interpretation of the results should be done with caution. Further trials with larger sample sizes, a control group, and long-term follow-up are required to confirm the effectiveness and safety of using rosemary as a strategy for reducing cardiovascular risk.

## CONCLUSION

Oral administration of dry extract capsules of *Rosmarinus officinalis L*. (1g/day) proved effective in reducing the estimated cardiovascular risk over 10 years in emergency department nursing professionals, demonstrating its potential for the treatment and control of cardiovascular risk factors. A reduction in systolic and diastolic blood pressure was identified, as well as a decrease in total cholesterol and high-density lipoprotein cholesterol levels, with an atheroprotective effect and few adverse events. However, the intervention time and/or the dose used may not have been sufficient to improve the glycemic profile and produce a clinically significant effect on FRS and blood pressure. The study’s limitations and the underexplored nature of the topic necessitate further research aimed at confirming the effectiveness and safety of using rosemary for cardiovascular health.

## Data Availability

The entire dataset supporting the results of this study is available upon request to the corresponding author.
