# Supplementary material for: Effectiveness of rosemary extract on the cardiovascular risk of emergency nursing professionals – an intervention study
Source: Rev Esc Enferm USP. 2026 Mar 16;60:e20250167. doi: 10.1590/1980-220X-REEUSP-2025-0167en (PMC13007777; doi:10.1590/1980-220X-REEUSP-2025-0167en)
Supplement: Translation

# Translation: Efetividade do extrato de alecrim sobre o risco cardiovascular de profissionais de enfermagem de emergência – estudo de intervenção*

**DOI:** 10.1590/1980-220X-REEUSP-2025-0167pt

**Authors:** Carolina Renz Pretto, Rosângela Marion da Silva, Juliana Tamiozzo, Luciane Flores Jacobi, Matias Nunes Frizzo, Christiane de Fátima Colet, Flávia Camef Dorneles Lenz, Silviamar Camponogara

**Affiliations:** 1 Secretaria Municipal de Saúde de Ijuí, Ijuí, RS, Brasil.; 2 Universidade Federal de Santa Maria, Santa Maria, RS, Brasil.; 3 Universidade Regional do Noroeste do Estado do Rio Grande do Sul, Ijuí, RS, Brasil.

**Keywords:** Rosmarinus, Terapias Complementares, Doenças Cardiovasculares, Fatores de Risco de Doenças Cardíacas, Enfermagem de Emergência

## Abstract

**Objetivo:**

Analisar a efetividade da administração oral de cápsulas de extrato seco de alecrim (*Rosmarinus officinalis L*.) (1g/dia) sobre a estimativa de risco cardiovascular, em dez anos, em profissionais de enfermagem de serviços de urgência e emergência.

**Método:**

Estudo quase-experimental desenvolvido no Sul do Brasil. Os participantes preencheram um questionário sociodemográfico e clínico, coletaram sangue para análise do perfil glicídico e lipídico e fizeram uso oral de cápsulas com 500 mg de extrato seco de alecrim duas vezes ao dia por 8 semanas; após, nova coleta de sangue foi realizada. O Escore de Risco Global de Framingham foi usado para estimar o risco cardiovascular. Análise de dados com estatística descritiva e inferencial; nível de significância de 5%.

**Resultados:**

Concluíram o estudo 36 profissionais, predominaram obesos e aqueles com colesterol total aumentado. Na comparação das variáveis, antes e depois da intervenção, verificaram-se diferenças na pressão arterial (p = 0,048), no colesterol total (p < 0,001) e na pontuação do Escore de Risco Global de Framingham (p = 0,047).

**Conclusão:**

O extrato seco de alecrim foi efetivo na redução da estimativa de risco cardiovascular dos profissionais de enfermagem. Registro Brasileiro de Ensaios Clínicos nº RBR 88hrnnw.

## INTRODUÇÃO

A morte por doenças cardiovasculares é um alerta para os sistemas de saúde. Representa 33% de todas as mortes, principalmente por infarto do miocárdio e acidente vascular cerebral. As causas envolvem um conjunto de fatores socioeconômicos, ambientais, comportamentais e genéticos, como dieta não saudável, estresse, sedentarismo, hipertensão arterial, diabetes, obesidade, sexo, idade e outros^(1)^. O risco cardiovascular pode ser determinado a partir do Escore de Risco Global de Framingham (ERGF), que estima a ocorrência de eventos coronarianos, cerebrovasculares, doença arterial periférica ou outros em 10 anos^(2)^.

Nas Unidades de Pronto Atendimento (UPA), o trabalho da equipe de enfermagem é caracterizado pelo atendimento rápido a pacientes com quadros agudos ou crônicos agudizados, que podem resultar em sofrimento, sequelas ou morte^(3)^. Esse contexto exige habilidade e conhecimento técnico-científico para realizar atividades que envolvem acolhimento, classificação de risco, estabilização, encaminhamento, gerenciamento, educação e pesquisa^(3)^. Essas características organizacionais e o processo de trabalho tornam os profissionais de enfermagem que atuam nesses serviços especialmente vulneráveis ao desenvolvimento de agravos à saúde. Portanto, trata-se de uma população com maior risco para essas doenças, particularmente devido à pressão assistencial, demandas físicas e emocionais intensas e ritmo acelerado de trabalho. Um estudo identificou que 52,9% dos profissionais de enfermagem de uma UPA apresentavam sobrepeso ou obesidade, 61,8% tinham circunferência abdominal aumentada, 58,8% eram sedentários, 79,4% relataram estresse moderado a altíssimo e 82,3% apresentavam má qualidade do sono^(4)^. Essas evidências reforçam a relevância de investigar condições de saúde nesse grupo.

Esse cenário do cuidado apresenta condições de trabalho inadequadas, relacionamento prejudicial, falta de recursos materiais e humanos, desvalorização profissional, sofrimento psíquico^(5)^; organização do trabalho em turnos, profissionais com má qualidade do sono e alteração nos níveis de cortisol^(6)^. Considerando tais aspectos, pesquisa mostrou relação entre o trabalho em turnos e aumento da pressão arterial, colesterol total e índice de massa corporal, e apontou o trabalho em turnos como fator de risco de doença arterial coronariana^(7)^. Outro estudo evidenciou associação entre níveis elevados de cortisol capilar e infarto agudo do miocárdio^(8)^.

A busca pelo bem-estar tem requerido abordagens terapêuticas complementares às convencionais, inclusive com uso de plantas medicinais. O uso de plantas medicinais não é recente, mas foi a partir de 1978 que a Organização Mundial da Saúde passou a reconhecê-las como recurso terapêutico e vem estimulando a formulação de políticas para sua utilização na atenção à saúde^(9)^, o que tem exigido mais pesquisas. No Brasil, a Política Nacional de Práticas Integrativas e Complementares no SUS (PNPIC), implantada em 2006 e atualizada em 2018^(10)^ e a Política Nacional de Plantas Medicinais e Fitoterápicos (PNPMF), instituída em 2006, têm ampliado o uso e o acesso a essas terapias^(11)^. O alecrim (*Rosmarinus officinalis L*.) é uma planta aromática, de natureza multifacetada e aplicações versáteis, constituída de diversos compostos ativos, diterpenos fenólicos, flavonoides e óleo essencial, com propriedades anti-inflamatórias e antioxidantes^(12)^ que merecem ser mais bem exploradas como terapêutica.

A literatura aponta número reduzido de pesquisas que avaliaram o efeito do alecrim sobre fatores de risco cardiovascular. Pesquisa com pacientes com diabetes mellitus evidenciou que a ingestão de chá de alecrim reduziu a medida da cintura e do quadril, a porcentagem de hemoglobina glicada e a resistência à insulina entre os participantes^(13)^. Outro estudo, com pessoas saudáveis e em tratamento para diabetes mellitus, mostrou redução nos níveis de glicose sanguínea e aumento na concentração de vitamina B12 em ambos os indivíduos^(14)^. Em pessoas saudáveis, investigação constatou diminuição no nível de glicose no sangue com ingesta de pó de alecrim e redução nos valores de lipídeos sanguíneos^(15)^.

Considerando o contexto de trabalho dos profissionais de enfermagem de emergência e a restrita evidência disponível sobre o alecrim e seu potencial terapêutico, a necessidade de produtos seguros para a redução do risco cardiovascular e morbimortalidade por doenças cardiovasculares, foi objetivo deste estudo analisar a efetividade da administração oral de cápsulas de extrato seco de alecrim (*Rosmarinus officinalis L*.) (1g/dia) sobre a estimativa de risco cardiovascular, em dez anos, em profissionais de enfermagem de serviços de urgência e emergência.

## MÉTODO

### Desenho e Local do Estudo

Trata-se de um estudo de intervenção, quase-experimental, com um único grupo pré e pós-teste, que seguiu as recomendações do *Consolidated Standards of Reporting Trials* (CONSORT). O estudo envolveu profissionais de enfermagem atuantes em serviços de emergência de uma cidade do interior do estado do Rio Grande do Sul, Brasil, a saber, uma Unidade de Pronto Atendimento e um Pronto-Socorro Hospitalar, que totalizavam 100 profissionais, entre enfermeiros e técnicos de enfermagem, sob qualquer forma de contratação. A UPA atende todo tipo de demanda e realiza cerca de 4,8mil atendimentos mensais; já o serviço de emergência hospitalar é referência em saúde na região onde se situa, considerado centro de atendimento de alta complexidade, e abrange uma população de aproximadamente 1,5 milhão de pessoas.

### Participantes

Entre os interessados em participar do estudo, excluíram-se aqueles com hipersensibilidade ao alecrim ou componentes da formulação fitoterápica, gestantes, lactantes, epilépticos, aqueles em tratamento para dislipidemia, com diagnóstico de diabetes mellitus, disfunção biliar ou hepática, doenças prostáticas ou gastroenterite e os afastados do trabalho por qualquer razão durante o período de coleta de dados. Os critérios de amostragem foram estabelecidos considerando as recomendações da Agência Nacional de Vigilância Sanitária sobre o uso de alecrim^(16)^ e para evitar possíveis confusões entre os resultados dos tratamentos para doenças pré-existentes e os efeitos do alecrim sobre os desfechos analisados.

### Tamanho da Amostra

Para o estabelecimento da amostra, foi considerada uma diferença de 2 pontos entre os valores do ERGF, antes e depois da intervenção; chance de 80% do erro tipo II (poder do teste); 5% de significância; e desvio padrão igual a 3,2, conforme estudo com enfermeiras de um hospital universitário^(17)^. Em virtude de possíveis perdas, foi acrescido 20% à amostra mínima. Desse modo, o quantitativo amostral estabelecido foi de 50 trabalhadores.

### Coleta de Dados

A coleta de dados ocorreu de agosto a novembro de 2022 e foi operacionalizada no local de trabalho dos participantes, em sala reservada. Foram coletados dados sociodemográficos: idade, sexo biológico, cor de pele autodeclarada, renda familiar, estado civil, escolaridade, cargo, turno de trabalho e carga horária; variáveis clínicas: circunferência abdominal (em centímetros), pressão arterial (em mmHg) e tabagismo.

A circunferência abdominal (CA) foi mensurada com o participante em pé, em posição ereta, com as mãos junto ao corpo, utilizando fita métrica para medição de circunferência corporal, com capacidade de 1,5 m/0,1 cm, posicionada na altura do umbigo com o abdômen relaxado, após expiração. Foram considerados como referência para a CA os parâmetros que caracterizam como obesidade central para a população da América do sul medida > 90 cm para homens e > 84 cm para mulheres.

As verificações da pressão arterial (PA) foram realizadas manualmente, com o participante sentado, uso de esfigmomanômetro aneroide calibrado, posicionado no membro superior direito apoiado a uma braçadeira, perímetro braquial elevado na altura do coração, com pernas descruzadas, pés apoiados no chão, bexiga vazia, e estetoscópio. Foram realizadas duas aferições pressóricas no mesmo braço, a primeira no início do turno de trabalho e outra um minuto após a primeira.

A partir das verificações, foi calculada a média da Pressão Arterial Sistólica (PAS) e da Pressão Arterial Diastólica (PAD) com o objetivo de se chegar o mais próximo possível dos valores reais da PA dos participantes. A PA foi classificada segundo a Atualização da Diretriz de Prevenção Cardiovascular da Sociedade Brasileira de Cardiologia^(2)^, na qual valores de PAS superiores a 120 mmHg e de PAD superiores a 80 mmHg são considerados elevados.

Coletas de sangue foram feitas para mensuração da dosagem de colesterol total (CT), Colesterol de Lipoproteína de Alta Densidade (HDL-c), e Hemoglobina Glicada (HbA1c). Foram considerados desejáveis, em jejum, valores de colesterol total menores que 190 mg/dL e de HDL-c superiores a 40 mg/dL. Os valores de HbA1c inferiores a 5,7% foram classificados como normais e ≥ 5,7%, alterados.

No dia anterior à coleta de sangue os participantes foram orientados por mensagem eletrônica, por meio de um aplicativo, a manter um jejum prévio de 12 horas. Foram coletados 5 mL de sangue na veia braquial, por profissional capacitado do laboratório contratado, transferidos para tubo vacutainer sem anticoagulante para análise dos parâmetros bioquímicos e submetidos a automação Cobas Integra 400, com insumos Roche.

A estratificação do risco cardiovascular foi realizada a partir do ERGF para homens e mulheres, que estima a ocorrência de eventos cardiovasculares em 10 anos^(2)^. Neste método, são considerados dados sobre sexo, idade, concentração de HDL-c, de CT, tabagismo, diabete mellitus (DM) e pressão arterial. Cada dado recebe uma pontuação conforme os valores apresentados por cada indivíduo, que somados, permitem o estabelecimento do percentual de risco cardiovascular. São considerados como de baixo risco indivíduos com ERGF <10%, risco médio 10%–20% e alto risco >20%.

O ERGF começa a pontuar a idade a partir dos 30 anos, ou seja, a faixa de 30 – 34 anos recebe pontuação zero. Considerando a limitada população desse estudo e para se evitar a exclusão de participantes, todos aqueles com idade inferior a 30 anos também receberam pontuação zero.

### Intervenção

A intervenção ocorreu após a primeira coleta de sangue e preenchimento dos demais instrumentos de coleta de dados. Todos os participantes receberam dois frascos contendo o total de 56 cápsulas com 500 mg de extrato seco de alecrim cada, para utilizar duas vezes ao dia (dose total de 1g/dia), por um período de oito semanas. Foi recomendada a ingestão da primeira cápsula pela manhã, 30 minutos depois do café da manhã, e a outra, 12 horas após a primeira, com água. Para aqueles que não costumavam tomar café da manhã, foi solicitada a ingestão 30 minutos após acordar.

As cápsulas foram produzidas por uma Farmácia Escola, contratada para esta função. Para a produção das cápsulas foi utilizado *Rosmarinus officinalis L*. extrato seco P. S. 0,03%, composto principalmente por pineno, canfeno, cineol, borneol, acetato de bornila, cânfora, diterpenos, saponina, traços de alcaloides, princípios amargos e taninos. O extrato seco P. S. 0,03% de *Rosmarinus officinalis L*. teve origem das folhas da planta; aspecto de pó fino higroscópico, sem materiais estranhos; cor bege esverdeado; odor e sabor característico; cinzas totais em valores inferiores a 5,0%; densidade entre 0,300 e 0,600; pH entre 4,0 e 8,0; parcialmente solúvel em água; umidade menor que 10,0%; teor ativo de flavonoides maior que 0,03; contagem de bactérias aeróbias inferior a 10.000, de fungos ou leveduras menor que 1.000 e de enterobactérias inferior a 1.000. As cápsulas foram confeccionadas com gelatina, sem cheiro ou sabor, preenchidas com 500 mg de extrato seco de alecrim e amido (para completar seu enchimento, em caso de necessidade).

Para maior segurança e controle quanto à adesão ao tratamento proposto e identificação de eventos adversos, disponibilizou-se a cada participante um mini diário para registro do horário das doses autoadministradas, das esquecidas ou usadas atrasadas e de possíveis reações e um infográfico com orientações sobre: manutenção do uso dos medicamentos de rotina, hábitos alimentares e de atividade física; doses e horários de autoadministração do fitoterápico; o que fazer diante do esquecimento da dose ou reação adversa; cuidados para a conservação; e contato dos pesquisadores. Todos os participantes foram orientados a guardar a cápsula que não foi ingerida, em casos de esquecimento, a fim de devolvê-las aos pesquisadores ao término do período de intervenção, como forma de controle das doses ingeridas.

Durante o período de intervenção, semanalmente, os participantes foram contatados via aplicativo de mensagens para acompanhamento da intervenção, incentivo ao uso do fitoterápico e da manutenção dos tratamentos de rotina.

Em relação às perdas de seguimento, foram considerados os participantes que desistiram de integrar a pesquisa antes de completar oito semanas de uso da intervenção e aqueles que mesmo após terem completado o esquema de doses se recusaram a realizar os exames laboratoriais ou não preencheram os questionários autoaplicáveis.

### Análise e Tratamento Dos Dados

Para a análise dos dados, foi considerado como desfecho primário o ERGF e como desfechos secundários IMC, CA, PAS, PAD, HbA1c, CT, LDL-c e HDL-c. Os dados coletados formaram um banco no programa Microsoft Excel^®^, construído a partir de dupla digitação independente e analisados com auxílio do software SPSS, versão 21.0 e do software Jamovi, versão 2.3.26.

As variáveis qualitativas foram expressas em frequências absolutas e relativas; as quantitativas, apresentadas como média e desvio padrão na presença de distribuição normal, ou mediana e intervalo interquartil na presença de distribuição assimétrica. A normalidade dos dados foi verificada por meio do teste de Shapiro-Wilk. A comparação das variáveis quantitativas antes e depois da intervenção ocorreu por meio do Teste t de Student para amostras pareadas, considerando uma distribuição normal, ou Wilcoxon, no caso de distribuição assimétrica. Valores de p < 0,05 foram considerados como estatisticamente significativos.

Como medidas de tamanho de efeito, utilizou-se d de Cohen (d) juntamente com Teste t de Student para amostras pareadas ou correlação bisserial de ordens, aliada ao Teste de Wilcoxon. Classificados como efeito pequeno, valores de d de Cohen iguais a 0,2; médio quando d = 0,5; e grande, d = 0,8. Para a correlação bisserial, considerou-se efeito pequeno, médio e grande, os valores 0,10, 0,24 e 0,37, respectivamente^(18)^.

A Análise de Variância de efeito misto (ANOVA mista) foi utilizada para comparar diferenças entre grupos pareados a partir de categorias não pareadas. Valores de p < 0,05 foram considerados estatisticamente significativos. A mensuração do tamanho do efeito foi realizada a partir do Eta quadrado generalizado (n2G), para o qual valores iguais a 0,01 são classificados como pequeno, iguais a 0,06 médio e 0,140 grande^(18)^.

### ASPECTOS Éticos

Todos os pressupostos da Declaração de Helsinque de 1984 e da Resolução nº 466/2012, que dispõem sobre pesquisas com pessoas no Brasil, foram seguidos. O projeto de pesquisa foi aprovado pelo Comitê de Ética em pesquisa da Universidade Federal de Santa Maria sob parecer n. 5.197.916 e foi registrado no Registro Brasileiro de Ensaios Clínicos – Rebec, nº RBR 88hrnnw. O consentimento livre e esclarecido foi obtido dos participantes na primeira e segunda etapa de coleta de dados.

## RESULTADOS

Da população convidada para integrar o estudo (n = 100), 36 recusaram-se a participar e 12 não atenderam aos critérios de seleção. Após o início da coleta de dados com 52 participantes, três não retornaram os questionários autoaplicáveis e um recusou-se a realizar a coleta de sangue e foram excluídos (Figura 1).

**Figura 1:** Fluxograma de constituição da amostra de profissionais de enfermagem que integrou o estudo quase-experimental.

Fonte: Adaptado do CONSORT 2010 Flow Diagram.

O início da intervenção ocorreu com 48 participantes. Passadas quatro semanas de intervenção, 12 profissionais interromperam o tratamento e não participaram da última fase de coleta de dados. A amostra final foi constituída por 36 participantes. A interrupção do uso do fitoterápico foi justificada pelos profissionais por sonolência, taquicardia, ansiedade, irritabilidade, cefaleia, dor estomacal/azia, razões particulares e gestação. Um participante relatou melhora na pele do rosto e manteve a terapia até o final.

Em relação às características dos profissionais, predominou o sexo biológico feminino, a idade < 40 anos, a cor branca e ter companheiro (Tabela 1). Quanto às características laborais, a maior parte era de nível médio, mas com formação superior, atuavam em turno rotativo e com carga horária > 44 horas semanais.

**Tabela 1:** Características sociolaborais de profissionais de enfermagem de serviços de emergência – Ijuí, RS, Brasil 2022 (n = 36).

Variáveis sociolaborais	Frequência	
	N	%
Sexo biológico
Masculino	6	16,7
Feminino	30	83,3
**Idade**		
≤ 40 anos	22	61,1
> 40 anos	14	38,9
**Cor de pele autodeclarada**		
Branca	30	83,3
Outra	6	16,7
**Estado civil**		
Com companheiro	26	72,2
Sem companheiro	10	27,8
**Renda familiar mensal**		
≤ 5.038 reais (≤ US$ 1618,68)	13	36,1
> 5.038 reais (> US$ 1618,68)	23	63,9
**Escolaridade**		
Ensino médio	11	30,6
Superior ou pós-graduação	25	69,4
**Cargo**		
Técnicos e auxiliares de enfermagem	28	77,8
Enfermeiro	8	22,2
**Turno de trabalho**		
Fixo	9	25,0
Rotativo	27	75,0
**Carga horária**		
≤ 44 h semanais	6	16,7
> 44 h semanais	30	83,3
**Total**	36	100,0

Quanto às variáveis clínicas, predominaram os profissionais de enfermagem obesos, com pressão arterial normal, colesterol total aumentado e com níveis desejáveis de HDL-c (Tabela 2).

**Tabela 2:** Variáveis clínicas e parâmetros laboratoriais de profissionais de enfermagem que atuam em serviços de emergência – Ijuí, RS, Brasil, 2022 (n = 36)

Variáveis clínicas e parâmetros laboratoriais	Frequência	
	N	%
**Circunferência abdominal**		
Normal	8	22,2
Obesidade central	28	77,8
**Pressão arterial**		
Normal	26	72,2
Elevada	10	27,8
**Uso de anti-hipertensivo**		
Sim	4	11,1
Não	32	88,9
**Tabagismo**		
Sim	4	11,1
Não	32	88,9
**Hemoglobina Glicada**		
Normal	27	75,0
Alterada	9	25,0
**Colesterol total**		
Desejável	14	38,9
Elevado	22	61,1
**Colesterol da Lipoproteína de Alta Densidade**		
Desejável	32	88,9
Diminuído	4	11,1

Na comparação das variáveis clínicas e parâmetros laboratoriais dos participantes antes e depois da intervenção com alecrim, verificaram-se diferenças significativas nas variáveis pressão arterial e colesterol (Tabela 3).

**Tabela 3:** Efetividade do alecrim sobre variáveis clínicas, comparação antes e após a intervenção – Ijuí, RS, Brasil, 2022 (n = 36).

Variáveis clínicas e parâmetros laboratoriais	Intervenção com alecrim		Tamanho do efeito	p-valor
	Antes Média ± DP	Depois Média ± DP
Circunferência Abdominal (cm)	98,08 ± 16,80	97,38 ± 15,38	0,091^a^	0,637^**^
Pressão arterial sistólica (mmHg)	117,03 ± 11,10	113,28 ± 13,39	0,342^b^	0,048^*^
Pressão arterial diastólica (mmHg)	78,31 ± 7,68	74,36 ± 9,10	0,423^b^	0,016^*^
Colesterol total (mg/dL)^c^	200,62 ± 29,87	185,25 ± 25,40	0,649^a^	<0,001^**^
Colesterol da Lipoproteína de alta densidade (mg/dl)	71,69 ± 24,11	57,41 ± 19,38	1,03^b^	<0,001^*^
Hemoglobina glicada (%)	5,43 ± 0,42	5,42 ± 0,33	0,054^b^	0,746^*^

Notas: *Teste T para amostras pareadas;

**Teste de Wilcoxon;

^a^Correlação bisserial de ordens;

^b^D de Cohen;

^c^n = 35.

Identificou-se redução significativa na média da pressão arterial sistólica e diastólica, com tamanho de efeito entre pequeno e médio. Salienta-se que a PAS se manteve inalterada em 16,7% (n = 6), apresentou redução em 50,0% (n = 18) e aumentou em 33,3% (n = 12). A pressão diastólica manteve-se constante em 25,0% (n = 9), diminuiu em 55,6% (n = 20) e aumentou em 19,4% (n = 7) dos profissionais.

Os parâmetros de CT tiveram diminuição significativa de efeito grande; entre os profissionais de enfermagem, 74,3% (n = 26) apresentaram redução após o uso de alecrim e 25,7% (n = 9) deles, aumento. Os níveis de HDL-c diminuíram significativamente, com tamanho de efeito grande. Houve redução na dosagem sérica de HDL-c em 86,1% (n = 31) dos profissionais, aumento em 11,1% (n = 4) e 2,8% (n = 1) mantiveram os valores constantes.

Na análise do ERGF, identificou-se mediana global de 3,5 pontos (IIQ = 6,00); 7,5 (IIQ = 11,5) para homens e 2 (IIQ = 7,0) para mulheres, com diferença entre os sexos (U = 34,50, p = 0,018). Para análise do efeito do alecrim sobre o ERGF, consideraram-se os fatores sexo e idade (Tabela 4). Contatou-se que profissionais do sexo biológico masculino têm maior risco para eventos cardiovasculares em 10 anos (p < 0,001), assim como aqueles com idade > 40 anos (p < 0,001), com tamanho de efeito grande.

**Tabela 4:** Efetividade do alecrim sobre a pontuação do Escore de Risco Global de Framingham, comparação entre os períodos de intervenção, de acordo com o sexo e a idade – Ijuí, RS, Brasil, 2022 (n = 35).

	Período	Sexo biológico		p-valor^*^ efeito do sexo (n^2^G)	p-valor^*^ interação intervenção-sexo (n^2^G)	Idade		p-valor^*^ efeito da idade (n^2^G)	p-valor^*^ interação intervenção-idade (n^2^G)	p-valor^*^ efeito da intervenção (n^2^G)	p-valor interação intervenção-sexo-idade (n^2^G)
		Masculino Média ± DP	Feminino Média ± DP			≤ 40 anos Média ± DP	> 40 anos Média ± DP				
**ERGF**	Antes	9,16 ± 6,85	2,31 ± 4,00	**<0,001** (0,382)	0,970 (0,00)	0,77 ± 2,99	8,07 ± 4,94	**<0,001** (0,474)	0,063 (0,015)	**0,047** (0,018)	0,440 (0,003)
	Depois	8,17 ± 5,19	1,45 ± 3,65			0,41 ± 3,06	6,31 ± 4,57				

Nota: ERGF – Escore de Risco Global de Framingham; DP – Desvio Padrão;

*ANOVA de efeito misto e tamanho de efeito - Eta quadrado generalizado (n^2^G).

A intervenção com alecrim reduziu a pontuação do ERGF significativamente (p = 0,047); contudo, o tamanho do efeito foi pequeno (n2G = 0,018). Destaca-se que 28,6% (n = 10) dos profissionais mantiveram o ERGF inalterado entre os períodos de observação, 51,4% (n = 18) apresentaram diminuição e 20,0% (n = 2) aumento.

## DISCUSSÃO

O principal resultado deste estudo é que a ingestão de cápsulas de extrato seco de *Rosmarinus officinalis L*. (1g/dia) pode colaborar na redução do risco para eventos cardiovasculares de profissionais de enfermagem de serviços de emergência, principalmente por favorecer a diminuição da pressão arterial e do colesterol total e com poucos efeitos adversos, mas os achados são preliminares e devem ser interpretados com cautela. É reconhecido que as DCVs são a principal causa de morbimortalidade no Brasil e no mundo, e entre seus fatores de risco destacam-se a dislipidemia e a hipertensão^(19)^; e fitoterápicos efetivos na redução desses fatores podem produzir impactos positivos.

Os resultados contribuem para a consolidação da ciência da enfermagem; ampliam as evidências sobre a utilização do alecrim no tratamento de fatores de risco para aterosclerose e doenças cardiovasculares, pouco exploradas até o momento; contribuem para o desenvolvimento científico baseado na diversidade cultural; validam o conhecimento popular sobre os benefícios do alecrim; e estimulam o protagonismo da enfermagem nas pesquisas com fitoterápicos.

Os achados desta investigação partiram de uma amostra na qual as características sociolaborais quanto à idade e ao sexo assemelham-se ao perfil da enfermagem no mundo, que é predominantemente feminino e relativamente jovem^(20)^. O nível educacional deles, com prevalência de educação superior ou maior, mesmo que a maioria dos profissionais possuía cargo de nível médio, reflete as mudanças no mercado de trabalho da enfermagem, que exige profissionalização, especialização e estimula a competitividade^(21)^.

A renda familiar mensal média de aproximadamente US$ 1618,68 (± US$ 677,15) ou R$ 5.838,00 permitiu classificar a enfermagem na classe média^(22)^, o que parece ser um bom indicador; contudo, verificou-se que a maioria dos profissionais cumpria carga horária de trabalho superior a 44 h semanais e atuava em turnos rotativos, com risco à sua saúde. Nesse sentido, estudo identificou associação entre o trabalho diurno com retorno ao serviço em menos de 11 h de forma rotineira e o risco para IAM^(23)^.

Verificou-se que quantitativo superior a 50% dos profissionais apresentava sobrepeso ou obesidade, circunferência abdominal aumentada, alterações nos níveis de colesterol total e quase 30% estava com alterações na pressão arterial sistêmica. Estes fatores de risco podem estar relacionados à organização do trabalho de enfermagem. Pesquisa reforça esta afirmativa indicando associação entre o trabalho em turnos com pressão arterial, colesterol total e IMC aumentados^(7)^. Da mesma forma, a desregulação na secreção de cortisol e o estresse crônico, comum entre estes profissionais, estão atrelados às mudanças epigenéticas envolvidas na dislipidemia, resistência insulínica, adiposidade, resposta imunológica prejudicada e aumento da inflamação sistêmica^(24)^.

Apesar desse contexto, os participantes apresentaram baixo risco para eventos cardiovasculares em 10 anos. Identificou-se relação entre a estimativa de risco cardiovascular e a idade e o sexo, maior em homens e naqueles com idade superior a 40 anos. Tal achado colabora para as evidências atuais de que a ocorrência de doenças cardiovasculares aumenta com o envelhecimento populacional e que a taxa mundial de mortalidade por DCV entre homens é maior comparada às mulheres^(1)^.

A idade cronológica é fator de risco, mas há interação e sinergia entre a idade cronológica e fatores de risco modificáveis no desenvolvimento de DCV, reforçando a importância de medidas preventivas e promotoras de envelhecimento saudável. Em relação ao sexo, entende-se que, embora haja prevalência menor de DCV em mulheres do que em homens, após um evento cardiovascular agudo as mulheres apresentam maior taxa de mortalidade e pior prognóstico. Também, destaca-se a influência da expressão genética dos cromossomos sexuais e das diferenças nos hormônios sexuais na fisiopatologia e ocorrência de eventos cardiovasculares^(25)^.

Em relação à efetividade do extrato seco de alecrim sobre os fatores de risco para as DCV, ao se comparar o período antes e após a intervenção, verificou-se redução significativa na pressão arterial sistólica e diastólica dos profissionais de enfermagem. Compostos do alecrim, como o ácido rosmarínico, que atuam na diminuição da angiotensina II e da enzima conversora de angiotensina relacionadas ao aumento da pressão arterial^(26)^, podem ter contribuído na redução da pressão arterial sistólica e diastólica dos participantes.

Considerando a elevada prevalência de hipertensão arterial na população e a relação de causalidade com a doença cardíaca isquêmica e mortalidade por acidente vascular encefálico, que repercutem em anos de vida perdidos por incapacidade^(19)^, o potencial hipotensor do alecrim pode colaborar na redução desses desfechos. Contudo, o efeito observado na amostra analisada foi de tamanho pequeno a médio, indicando importância clínico-prática limitada.

Na presente pesquisa, não se identificou diferença estatisticamente significativa entre os períodos de intervenção sobre a circunferência abdominal, embora tenha havido uma redução em seus valores médios, o que pode estar relacionado à dose utilizada e/ou ao tempo de intervenção. Estudo com mulheres obesas que envolveu ingestão de cápsulas com óleo de alecrim na dose de 380 mg/dia por seis semanas aliada à dieta mostrou redução da relação cintura-quadril^(27)^, assim como constatado em pessoas com DM que usaram chá de alecrim na dose de 2g/dia por 12 semanas^(13)^. Considerando que a relação cintura-quadril apresenta associação com excesso de peso, síndrome metabólica e DM, a redução desta medida, vinculada ao uso do alecrim, pode repercutir na redução do risco cardiovascular.

Os níveis de colesterol total também foram menores após a intervenção, com tamanho de efeito grande, sugerindo importância clínica. Achado semelhante foi verificado em estudo que avaliou o efeito de alecrim em pó nas doses de 5 e 10 g/dia por 8 semanas sobre o perfil lipídico de pessoas saudáveis^(15)^. O mecanismo de ação do alecrim, envolvido na diminuição do colesterol e triglicerídeos, está ligado à sua capacidade anti-inflamatória e antioxidante que interfere no metabolismo lipídico, inibe a lipólise, a maturação de adipócitos, reduz a síntese de colesterol e ácidos graxos no fígado e tecido adiposo e aumenta a sua depuração^(28)^.

A partir do mecanismo de ação do alecrim, pode-se inferir que os resultados da presente pesquisa reforçam as evidências sobre o potencial da planta para a saúde vascular, pois a participação do colesterol nos processos ateroscleróticos está relacionada à capacidade individual de absorção deste lipídeo, ao tipo de absorção que contribui para a composição e qualidade das partículas de LDL-c, e sua eliminação ineficaz^(29)^.

Referente ao HDL-c, na comparação entre os períodos de intervenção, contatou-se redução significativa do parâmetro, de efeito grande. Pesquisa tem sugerido que, antes da menopausa, níveis elevados de HDL-c são cardioprotetores para mulheres, mas não naquelas que estão em transição para a menopausa. A redução dos níveis de estradiol influencia em fatores que afetam a qualidade da partícula de HDL-c, sua composição e função, tornando-a um fator pró-inflamatório e pró-aterogênico, independentemente da idade, raça/etnia, educação, IMC, PAS, LDL-c, uso de medicamentos para DCV e proteína C reativa^(30)^.

Pesquisa realizada com adultos chineses evidenciou associação em forma de U entre o HDL-c e mortalidade por todas as causas, por DCV e câncer. Evidenciou-se que uma em cada trinta mortes por todas as causas pode ser atribuída a níveis elevados e baixos de HDL-c e que valores entre 50 a 79 mg/dL representam menor risco de mortalidade por todas as causas e por DCV. Altos e baixos níveis de HDL-c foram associados ao aumento de mortalidade por doença cardíaca isquêmica e doença isquêmica cerebrovascular. Os autores também apontam para o potencial inflamatório e aterogênico da molécula quando apresenta alterações na composição proteica e torna-se disfuncional^(31)^. Diante disso, sugere-se um efeito benéfico do alecrim sobre este desfecho, à medida que a média dos valores de HDL-c dos participantes se manteve na faixa de normalidade. Contudo, o efeito do alecrim sobre essa molécula deve ser investigado em longo prazo, considerando que a redução progressiva dos níveis de HDL-c pode ser danosa para a saúde cardiovascular e subsidiar questionamentos quanto à segurança da planta.

Não foram constatadas alterações significativas na hemoglobina glicada entre os períodos de intervenção, em concordância com o estudo com pessoas saudáveis e com diabetes^(14)^, no qual o uso de 3 g/dia de pó de *Rosmarinus oficinallis L.* encapsulado por 8 semanas por pessoas saudáveis não apresentou diferença significativa com a intervenção. Por outro lado, estudo sugere que o alecrim e seus compostos fenólicos atuam na melhora da resistência insulínica, supressão da gliconeogênese, estimulação da captação da glicose e têm efeito anti-inflamatório e antioxidante^(32)^, apontando a necessidade de investigações com maior tempo de intervenção ou com doses diferentes da pesquisada para esclarecimento da efetividade do alecrim sobre esse parâmetro.

Com a intervenção, identificou-se redução significativa na pontuação do ERGF, porém, com limitada importância clinico-prática. Destaca-se que 51,4% dos profissionais apresentaram diminuição da pontuação, o que pode estar associado à redução dos parâmetros de pressão arterial e colesterol total identificados nos participantes, considerando que o algoritmo leva em conta tais variáveis para estimar o risco^(2)^.

No mundo, mais de meio bilhão de pessoas são afetadas por doenças cardiovasculares, principalmente doença cardíaca isquêmica e acidente vascular encefálico, e os avanços médicos na área concentram-se em países de alta renda^(1)^. Pesquisas com intuito de fornecer evidências sobre uma terapia complementar ou adjuvante no tratamento dos fatores de risco para essas doenças de fácil acesso à população, em um país subdesenvolvido, é uma inovação com ganho rumo à redução da mortalidade prematura por doenças crônicas não transmissíveis, alvo dos Objetivos do Desenvolvimento Sustentável.

Ainda em relação à intervenção com o alecrim, a análise de sua efetividade sobre desfechos de saúde dos profissionais de enfermagem, com vistas à segurança dos participantes, ocorreu aliada ao acompanhamento e identificação de eventos adversos. Estiveram, possivelmente, relacionados à intervenção eventos como sonolência, melhora na pele do rosto, taquicardia, ansiedade, irritabilidade, choro frequente, cefaleia e dor estomacal/azia, diferente do que foi observado em estudo com pessoas privadas de liberdade^(33)^, que com intervenção semelhante não identificou eventos adversos. Estes achados reforçam a necessidade de mais pesquisas que avaliem sua segurança e efetividade em diferentes desfechos. Eles sinalizam a importância de ações de promoção do uso racional e de educação para uso da preparação botânica correta, respeito à dosagem, via de administração e período de uso e desmistificação de inocuidade.

Diferentes preparações botânicas, como por exemplo extrato seco e óleo essencial, podem provocar efeitos diferentes no organismo, assim como a concentração de determinados compostos químicos nessas substâncias. Um exemplo disso foi o resultado apresentado por pesquisa com alecrim direcionado a pessoas com diagnóstico de hipotensão primária, na qual 1 ml de óleo essencial em um torrão de açúcar a cada 8 horas por 44 semanas mostrou tendência ao aumento da pressão arterial^(34)^, diferente do efeito hipotensor encontrado nesse estudo com o extrato seco com teor ativo de flavonoides maior que 0,03. É importante destacar que o óleo essencial é um produto aromático, volátil, um metabólito secundário utilizado pelas plantas como proteção, cuja eficácia depende da concentração e interação sinérgica de seus constituintes químicos, influenciada pelos processos de extração, ambiente de desenvolvimento da planta e outros^(35)^. O extrato seco, por sua vez, é uma preparação sólida obtida de drogas vegetais, resulta da evaporação do solvente utilizado no processo de extração e da eliminação de materiais indesejáveis e que conta com especificações de constituintes químicos (para fins de controle de qualidade)^(16)^.

A realização dessa pesquisa apresentou algumas limitações, entre elas: o pequeno tamanho da amostra devido às perdas de seguimento, que pode introduzir viés de atrito; o ERGF que tem baixa sensibilidade para prever eventos em curto prazo; a falta de monitoramento sistemático de variáveis comportamentais relevantes, como alimentação, prática de atividade física, uso concomitante de medicamentos e níveis de estresse ou qualidade do sono, com potencial viés de confundimento; a ausência de grupo controle; e a avaliação da efetividade do alecrim sobre a hemoglobina glicada com intervenção que durou menos de 3 meses, o que pode ter influenciado nos resultados. Ainda, o pequeno tamanho de efeito observado sobre o ERGF e a pressão arterial indica efeito clínico limitado para estes desfechos. Apesar disso, os resultados agregaram evidências com potencial de produzir impacto social, econômico e nos indicadores de morbimortalidade.

Diante das limitações e por se tratar de um estudo preliminar, a interpretação dos resultados deve ser feita com cautela. Novos ensaios com maior amostragem, grupo controle e seguimento em longo prazo são necessários para confirmar a efetividade e segurança do uso do alecrim como estratégia para a redução do risco cardiovascular.

## CONCLUSÃO

A administração oral de cápsulas de extrato seco de *Rosmarinus officinalis L*. (1g/dia) mostrou-se efetiva na redução da estimativa de risco cardiovascular, em 10 anos, de profissionais de enfermagem de serviços de emergência, evidenciando seu potencial para o tratamento e controle de fatores de risco cardiovasculares. Identificou-se redução da pressão arterial sistólica e diastólica; dos níveis de colesterol total e de colesterol da lipoproteína de alta densidade, com efeito ateroprotetor e poucos eventos adversos. Contudo, o tempo de intervenção e/ou a dose utilizada podem não ter sido suficientes para melhorar o perfil glicídico e produzir efeito clínico importante sobre o ERGF e pressão arterial. As limitações do estudo e a temática pouco explorada requerem mais pesquisas sobre o tema direcionadas à confirmação da efetividade e segurança do uso do Alecrim para a saúde cardiovascular.

## Data Availability

Todo o conjunto de dados que dá suporte aos resultados deste estudo está disponível mediante solicitação ao autor correspondente.

# Translation: Eficacia del extracto de romero sobre el riesgo cardiovascular de los profesionales de enfermería de urgencias – estudio de intervención^*^

**DOI:** 10.1590/1980-220X-REEUSP-2025-0167es

**Authors:** Carolina Renz Pretto, Rosângela Marion da Silva, Juliana Tamiozzo, Luciane Flores Jacobi, Matias Nunes Frizzo, Christiane de Fátima Colet, Flávia Camef Dorneles Lenz, Silviamar Camponogara

**Affiliations:** 1 Secretaria Municipal de Saúde de Ijuí, Ijuí, RS, Brasil.; 2 Universidade Federal de Santa Maria, Santa Maria, RS, Brasil.; 3 Universidade Regional do Noroeste do Estado do Rio Grande do Sul, Ijuí, RS, Brasil.

**Keywords:** Rosmarinus, Terapias Complementarias, Enfermedades Cardiovasculares, Factores de Riesgo de Enfermedad Cardiaca, Enfermería de Urgencia

## Abstract

**Objetivo:**

Analizar la efectividad de la administración oral de cápsulas de extracto seco de Romero (*Rosmarinus officinalis L*.) (1g/día) sobre el riesgo cardiovascular estimado, a lo largo de diez años, en profesionales de enfermería que trabajan en servicios de urgencias y emergencias.

**Método:**

Estudio cuasi-experimental realizado en el sur de Brasil. Los participantes completaron un cuestionario sociodemográfico y clínico, se les extrajo sangre para análisis de perfil glucémico y lipídico y tomaron cápsulas orales que contenían 500 mg de extracto de romero seco dos veces al día durante 8 semanas, después de lo cual, se les tomó una nueva muestra de sangre. Para estimar el riesgo cardiovascular se utilizó la escala de riesgo global de Framingham. Análisis de datos mediante estadística descriptiva e inferencial; nivel de significancia del 5%.

**Resultados:**

El estudio incluyó a 36 profesionales, predominantemente obesos y con colesterol total elevado. Al comparar las variables antes y después de la intervención, se encontraron diferencias en la presión arterial (p = 0,048), en el colesterol total (p < 0,001) y en la escala de riesgo global de Framingham (p = 0,047).

**Conclusión:**

El extracto seco de Romero fue eficaz para reducir el riesgo cardiovascular estimado en profesionales de enfermería. Registro Brasileño de Ensayos Clínicos No. RBR 88hrnnw.

## INTRODUCCIÓN

La muerte por enfermedades cardiovasculares es una alerta para los sistemas de salud. Representa el 33% de todas las muertes, principalmente por infarto de miocardio y accidente cerebrovascular. Las causas implican un conjunto de factores socioeconómicos, ambientales, conductuales y genéticos, como una dieta poco saludable, estrés, sedentarismo, hipertensión arterial, diabetes, obesidad, sexo, edad y otros^(1)^. El riesgo cardiovascular puede determinarse a partir de la puntuación de riesgo global de Framingham (ERGF), que estima la ocurrencia de eventos coronarios, cerebrovasculares, enfermedad arterial periférica u otros en 10 años^(2)^.

En las Unidades de Atención de Urgencias (UPA en portugués), el trabajo del equipo de enfermería se caracteriza por la atención rápida a pacientes con cuadros agudos o crónicos agudos, que pueden provocar sufrimiento, secuelas o la muerte^(3)^. Este contexto exige habilidad y conocimientos técnico-científicos para realizar actividades que implican la acogida, la clasificación de riesgos, la estabilización, la derivación, la gestión, la educación y la investigación^(3)^. Estas características organizativas y el proceso de trabajo hacen que los profesionales de enfermería que trabajan en estos servicios sean especialmente vulnerables al desarrollo de problemas de salud. Por lo tanto, se trata de una población con mayor riesgo de padecer estas enfermedades, sobre todo debido a la presión asistencial, las intensas exigencias físicas y emocionales y el ritmo acelerado de trabajo. Un estudio identificó que el 52,9% de los profesionales de enfermería de una UPA presentaban sobrepeso u obesidad, el 61,8% tenían un perímetro abdominal aumentado, el 58,8% eran sedentarios, el 79,4% informaron de un estrés moderado a muy alto y el 82,3% presentaban mala calidad del sueño^(4)^. Estas evidencias refuerzan la importancia de investigar las condiciones de salud de este grupo.

Este escenario de atención presenta condiciones de trabajo inadecuadas, relaciones perjudiciales, falta de recursos materiales y humanos, desvalorización profesional, sufrimiento psíquico^(5)^; organización del trabajo por turnos, profesionales con mala calidad del sueño y alteración de los niveles de cortisol^(6)^.

Teniendo en cuenta estos aspectos, la investigación mostró una relación entre el trabajo por turnos y el aumento de la presión arterial, el colesterol total y el índice de masa corporal, y señaló el trabajo por turnos como factor de riesgo de enfermedad arterial coronaria^(7)^. Otro estudio evidenció la asociación entre niveles elevados de cortisol capilar y el infarto agudo de miocardio^(8)^.

La búsqueda del bienestar ha requerido enfoques terapéuticos complementarios a los convencionales, incluyendo el uso de plantas medicinales. El uso de plantas medicinales no es reciente, pero fue a partir de 1978 que la Organización Mundial de la Salud comenzó a reconocerlas como recurso terapéutico y ha estado estimulando la formulación de políticas para su uso en la atención de la salud^(9)^, lo que ha exigido más investigaciones. En Brasil, la Política Nacional de Prácticas Integrativas y Complementarias en el Sistema Único de Salud (PNPIC), implantada en 2006 y actualizada en 2018^(10)^, y la Política Nacional de Plantas Medicinales y Fitoterápicos (PNPMF), instituida en 2006, han ampliado el uso y el acceso a estas terapias^(11)^. El romero (*Rosmarinus officinalis L*.) es una planta aromática, de naturaleza multifacética y aplicaciones versátiles, compuesta por diversos compuestos activos, diterpenos fenólicos, flavonoides y aceite esencial, con propiedades antiinflamatorias y antioxidantes^(12)^ que merecen ser mejor exploradas como terapia.

La literatura señala un número reducido de investigaciones que han evaluado el efecto del romero sobre los factores de riesgo cardiovascular. Una investigación con pacientes con diabetes mellitus evidenció que la ingesta de té de romero redujo la medida de la cintura y la cadera, el porcentaje de hemoglobina glicosilada y la resistencia a la insulina entre los participantes^(13)^. Otro estudio, con personas sanas y en tratamiento para la diabetes mellitus, mostró una reducción en los niveles de glucosa en sangre y un aumento en la concentración de vitamina B12 en ambos individuos^(14)^. En personas sanas, la investigación constató una disminución en el nivel de glucosa en sangre con la ingesta de polvo de romero y una reducción en los valores de lípidos en sangre^(15)^.

Teniendo en cuenta el contexto laboral de los profesionales de enfermería de urgencias y la escasa evidencia disponible sobre el romero y su potencial terapéutico, así como la necesidad de productos seguros para reducir el riesgo cardiovascular y la morbimortalidad por enfermedades cardiovasculares, el objetivo de este estudio fue analizar la eficacia de la administración oral de cápsulas de extracto seco de romero (*Rosmarinus officinalis L*.) (1 g/día) sobre la estimación del riesgo cardiovascular a diez años en profesionales de enfermería de servicios de urgencias y emergencias.

## MÉTODO

### Diseño y Lugar Del Estudio

Se trata de un estudio de intervención, cuasi-experimental, con un único grupo pre y post-test, que siguió las recomendaciones de las *Normas Consolidadas para la Presentación de Ensayos Clínicos* (CONSORT). El estudio involucró a profesionales de enfermería que trabajan en servicios de emergencia de una ciudad del interior del estado de Rio Grande do Sul, Brasil, a saber, una Unidad de Atención de Urgencias y un Servicio de Urgencias Hospitalarias, que sumaban un total de 100 profesionales, entre enfermeros y técnicos de enfermería, con cualquier tipo de contrato. La UPA atiende todo tipo de demandas y realiza alrededor de 4,800 atendimientos mensuales; por su parte, el servicio de urgencias hospitalarias es una referencia en salud en la región donde se encuentra, considerado un centro de atención de alta complejidad, y abarca una población de aproximadamente 1.5 millones de personas.

### Participantes

Entre los interesados en participar en el estudio, se excluyó a aquellos con hipersensibilidad al romero o a los componentes de la formulación fitoterapéutica, embarazadas, lactantes, epilépticos, aquellos en tratamiento por dislipidemia, con diagnóstico de diabetes mellitus, disfunción biliar o hepática, enfermedades prostáticas o gastroenteritis y los ausentes del trabajo por cualquier motivo durante el período de Recolección de datos.

Los criterios de muestreo se establecieron teniendo en cuenta las recomendaciones de la Agencia Nacional de Vigilancia Sanitaria sobre el uso del romero^(16)^ y para evitar posibles confusiones entre los resultados de los tratamientos para enfermedades preexistentes y los efectos del romero sobre los resultados analizados.

### Tamaño de la Muestra

Para establecer la muestra, se consideró una diferencia de 2 puntos entre los valores del ERGF antes y después de la intervención; una probabilidad del 80% de error de tipo II (potencia de la prueba); una significación del 5%; y una desviación estándar igual a 3,2, según un estudio realizado con enfermeras de un hospital universitario^(17)^. Debido a posibles pérdidas, se añadió un 20% a la muestra mínima. De este modo, la muestra cuantitativa establecida fue de 50 trabajadores.

### Recolección de Datos

La recolección de datos se llevó a cabo entre agosto y noviembre de 2022 y se realizó en el lugar de trabajo de los participantes, en una sala reservada. Se recopilaron datos sociodemográficos: edad, sexo biológico, color de piel autodeclarado, ingresos familiares, estado civil, nivel educativo, cargo, turno de trabajo y carga horaria; variables clínicas: circunferencia abdominal (en centímetros), presión arterial (en mmHg) y tabaquismo.

La circunferencia abdominal (CA) se midió con el participante de pie, en posición erguida, con las manos pegadas al cuerpo, utilizando una cinta métrica para medir la circunferencia corporal, con una capacidad de 1,5 m/0,1 cm, colocada a la altura del ombligo con el abdomen relajado, después de la espiración. Se consideraron como referencia para el CA los parámetros que caracterizan la obesidad central para la población de América del Sur, medidos > 90 cm para hombres y > 84 cm para mujeres.

Las mediciones de la presión arterial (PA) se realizaron manualmente, con el participante sentado, utilizando un esfigmomanómetro aneroide calibrado, colocado en el miembro superior derecho apoyado en un brazalete, con el perímetro braquial elevado a la altura del corazón, con las piernas descruzadas, los pies apoyados en el suelo, la vejiga vacía y un estetoscopio. Se realizaron dos mediciones de presión en el mismo brazo, la primera al inicio del turno de trabajo y la otra un minuto después de la primera.

A partir de las mediciones, se calculó el promedio de la presión arterial sistólica (PAS) y la presión arterial diastólica (PAD) con el objetivo de acercarse lo más posible a los valores reales de PA de los participantes. La PA se clasificó según la Actualización de la Guía de Prevención Cardiovascular de la Sociedad Brasileña de Cardiología^(2)^, en la que se consideran elevados los valores de PAS superiores a 120 mmHg y de PAD superiores a 80 mmHg.

Se realizaron recolecciones de sangre para medir los niveles de colesterol total (CT), colesterol de lipoproteínas de alta densidad (HDL-c) y hemoglobina glicosilada (HbA1c). Se consideraron deseables, en ayunas, valores de colesterol total inferiores a 190 mg/dL y de HDL-c superiores a 40 mg/dL. Los valores de HbA1c inferiores al 5,7% se clasificaron como normales y los ≥ 5,7%, como alterados.

El día anterior a la recolección de sangre, se indicó a los participantes mediante un mensaje electrónico, a través de una aplicación, que mantuvieran un ayuno previo de 12 horas. Se realizó la recolección de 5 ml de sangre de la vena braquial por parte de un profesional cualificado del laboratorio contratado, se transfirieron a un tubo vacutainer sin anticoagulante para el análisis de los parámetros bioquímicos y se sometieron a la automatización Cobas Integra 400, con insumos Roche.

La estratificación del riesgo cardiovascular se realizó a partir del ERGF para hombres y mujeres, que estima la ocurrencia de eventos cardiovasculares en 10 años^(2)^. En este método, se tienen en cuenta datos sobre sexo, edad, concentración de HDL-c, CT, tabaquismo, diabetes mellitus (DM) y presión arterial. Cada dato recibe una puntuación según los valores presentados por cada individuo, que sumados permiten establecer el porcentaje de riesgo cardiovascular. Se consideran de bajo riesgo los individuos con ERGF <10%, de riesgo medio 10%–20% y de alto riesgo >20%.

El ERGF comienza a puntuar la edad a partir de los 30 años, es decir, el rango de 30 a 34 años recibe una puntuación de cero. Teniendo en cuenta la limitada población de este estudio y para evitar la exclusión de participantes, todos los menores de 30 años también recibieron una puntuación de cero.

### Intervención

La intervención se llevó a cabo después de la primera recolección de sangre y de completar los demás instrumentos de recopilación de datos. Todos los participantes recibieron dos frascos con un total de 56 cápsulas de 500 mg de extracto seco de romero cada uno, para tomar dos veces al día (dosis total de 1 g/día), durante un periodo de ocho semanas. Se recomendó tomar la primera cápsula por la mañana, 30 minutos después del desayuno, y la otra, 12 horas después de la primera, con agua. A aquellos que no solían desayunar, se les pidió que la tomaran 30 minutos después de levantarse. Las cápsulas fueron producidas por una farmacia escuela, contratada para esta función.

Para la producción de las cápsulas se utilizó extracto seco de *Rosmarinus officinalis L.* P. S. 0,03%, compuesto principalmente por pineno, camfeno, cineol, borneol, acetato de bornilo, alcanfor, diterpenos, saponina, trazas de alcaloides, principios amargos y taninos. El extracto seco P. S. 0,03% de *Rosmarinus officinalis L*. se obtuvo de las hojas de la planta; aspecto de polvo fino higroscópico, sin materiales extraños; color beige verdoso; olor y sabor característicos; cenizas totales en valores inferiores al 5,0%; densidad entre 0,300 y 0,600; pH entre 4,0 y 8,0; parcialmente soluble en agua; humedad inferior al 10,0%; contenido activo de flavonoides superior al 0,03; recuento de bacterias aeróbicas inferior a 10 000, de hongos o levaduras inferior a 1000 y de enterobacterias inferior a 1000. Las cápsulas se fabricaron con gelatina, sin olor ni sabor, y se rellenaron con 500 mg de extracto seco de romero y almidón (para completar su relleno, en caso de necesidad).

Para mayor seguridad y control en cuanto al cumplimiento del tratamiento propuesto y la identificación de eventos adversos, se proporcionó a cada participante un mini diario para registrar la hora de las dosis autoadministradas, las olvidadas o utilizadas con retraso y las posibles reacciones, así como un infográfico con orientaciones sobre: el mantenimiento del uso de los medicamentos de rutina, los hábitos alimenticios y la actividad física; las dosis y los horarios de autoadministración del fitoterápico; qué hacer en caso de olvido de la dosis o reacción adversa; cuidados para la conservación; y contacto de los investigadores. Se indicó a todos los participantes que guardaran las cápsulas que no se hubieran ingerido, en caso de olvido, para devolverlas a los investigadores al final del periodo de intervención, como forma de control de las dosis ingeridas.

Durante el periodo de intervención, se contactó semanalmente con los participantes a través de una aplicación de mensajes para hacer un seguimiento de la intervención, fomentar el uso del fitoterápico y mantener los tratamientos rutinarios.

En cuanto a las pérdidas de seguimiento, se consideraron los participantes que abandonaron la investigación antes de completar ocho semanas de uso de la intervención y aquellos que, incluso después de haber completado el esquema de dosis, se negaron a realizar los exámenes de laboratorio o no completaron los cuestionarios autoaplicables.

### Análisis y Tratamiento de Los Datos

Para el análisis de los datos, se consideró como resultado primario el ERGF y como resultados secundarios el IMC, la CA, la PAS, la PAD, la HbA1c, el CT, el LDL-c y el HDL-c. Los datos recopilados se introdujeron en una base de datos en el programa Microsoft Excel®, construida a partir de una doble entrada independiente, y se analizaron con la ayuda del software SPSS, versión 21.0, y del software Jamovi, versión 2.3.26.

Las variables cualitativas se expresaron en frecuencias absolutas y relativas; las cuantitativas, se presentaron como media y desviación estándar en presencia de distribución normal, o mediana e intervalo intercuartílico en presencia de distribución asimétrica. La normalidad de los datos se verificó mediante la prueba de Shapiro-Wilk. La comparación de las variables cuantitativas antes y después de la intervención se realizó mediante la prueba t de Student para muestras emparejadas, considerando una distribución normal, o Wilcoxon, en caso de distribución asimétrica. Los valores de p < 0,05 se consideraron estadísticamente significativos.

Como medidas del tamaño del efecto, se utilizó la d de Cohen (d) junto con la prueba t de Student para muestras emparejadas o la correlación biserial de órdenes, junto con la prueba de Wilcoxon. Se clasificaron como efecto pequeño los valores de la d de Cohen iguales a 0,2; medio cuando d = 0,5; y grande, d = 0,8. Para la correlación biserial, se consideraron efectos pequeños, medianos y grandes los valores 0,10, 0,24 y 0,37, respectivamente^(18)^.

Se utilizó el análisis de varianza de efectos mixtos (ANOVA mixta) para comparar las diferencias entre grupos emparejados a partir de categorías no emparejadas. Los valores de p < 0,05 se consideraron estadísticamente significativos. La medición del tamaño del efecto se realizó a partir del Eta cuadrado generalizado (n2G), para el cual los valores iguales a 0,01 se clasifican como pequeños, los iguales a 0,06 como medianos y los iguales a 0,140 como grandes^(18)^.

### Aspectos Éticos

Se siguieron todos los supuestos de la Declaración de Helsinki de 1984 y de la Resolución n.º 466/2012, que regulan la investigación con personas en Brasil. El proyecto de investigación fue aprobado por el Comité de Ética en Investigación de la Universidad Federal de Santa María con el dictamen n.º 5.197.916 y se registró en el Registro Brasileño de Ensayos Clínicos (Rebec), n.º RBR 88hrnnw. Se obtuvo el consentimiento libre e informado de los participantes en la primera y segunda etapa de recolección de datos.

## RESULTADOS

De la población invitada a participar en el estudio (n = 100), 36 se negaron a participar y 12 no cumplieron los criterios de selección. Tras el inicio de la recolección de datos con 52 participantes, tres no devolvieron los cuestionarios autoaplicables y uno se negó a realizar la extracción de sangre, por lo que fueron excluidos (Figura 1).

**Figura 1:** Diagrama de flujo de la constitución de la muestra de profesionales de enfermería que integraron el estudio cuasi-experimental.

Fuente: Adaptado del Diagrama de Flujo CONSORT 2010.

El inicio de la intervención contó con 48 participantes. Tras cuatro semanas de intervención, 12 profesionales interrumpieron el tratamiento y no participaron en la última fase de recolección de datos. La muestra final estuvo compuesta por 36 participantes. Los profesionales justificaron la interrupción del uso del fitoterápico por somnolencia, taquicardia, ansiedad, irritabilidad, cefalea, dolor de estómago/acidez, razones particulares y embarazo. Un participante informó de una mejora en la piel del rostro y mantuvo la terapia hasta el final.

En cuanto a las características de los profesionales, predominaron el sexo biológico femenino, la edad < 40 años, la raza blanca y tener pareja (Tabla 1). En cuanto a las características laborales, la mayoría tenía estudios de nivel medio, pero con formación superior, trabajaban en turnos rotativos y con una carga horaria > 44 horas semanales.

**Tabla 1:** Características sociolaborales de los profesionales de enfermería de los servicios de urgencias – Ijuí, RS, Brasil 2022 (n = 36).

Variables sociolaborales	Frecuencia	
	N	%
**Sexo biológic**		
Masculino	6	16,7
Femenino	30	83,3
**Edad**		
≤ 40 años	22	61,1
> 40 años	14	38,9
**Color de piel (autodeclarado)**		
Blanca	30	83,3
Otra	6	16,7
**Estado civil**		
Con compañero	26	72,2
Sin compañero	10	27,8
**Ingreso familiar mensual**		
≤ 5.038 reales (≤ US$ 1618,68)	13	36,1
> 5.038 reales (> US$ 1618,68)	23	63,9
**Escolaridad**		
Enseñanza media	11	30,6
Superior o postgrado	25	69,4
**Cargo**		
Técnicos y auxiliares de enfermería	28	77,8
Enfermeros	8	22,2
**Turno de trabajo**		
Fijo	9	25,0
Rotativo	27	75,0
**Carga horaria**		
≤ 44 h semanales	6	16,7
> 44 h semanales	30	83,3
**Total**	36	100,0

En cuanto a las variables clínicas, predominaron los profesionales de enfermería obesos, con presión arterial normal, colesterol total elevado y niveles deseables de HDL-c (Tabla 2).

**Tabla 2:** Variables clínicas y parámetros de laboratorio de los profesionales de enfermería que trabajan en servicios de urgencias – Ijuí, RS, Brasil, 2022 (n = 36).

Variables clínicas y parámetros laboratoriales	Frecuencia	
	N	%
**Circunferencia abdominal**		
Normal	8	22,2
Obesidad central	28	77,8
**Presión arterial**		
Normal	26	72,2
Elevada	10	27,8
**Uso de anti-hipertensivo**		
Si	4	11,1
No	32	88,9
**Tabaquismo**		
Si	4	11,1
No	32	88,9
**Hemoglobina Glicosilada**		
Normal	27	75,0
Alterada	9	25,0
**Colesterol total**		
Deseable	14	38,9
Elevado	22	61,1
**Colesterol da Lipoproteína de Alta Densidad**		
Deseable	32	88,9
Disminuido	4	11,1

Al comparar las variables clínicas y los parámetros de laboratorio de los participantes antes y después de la intervención con romero, se observaron diferencias significativas en las variables de presión arterial y colesterol (Tabla 3).

**Tabla 3:** Eficacia del romero sobre las variables clínicas, comparación antes y después de la intervención – Ijuí, RS, Brasil, 2022 (n = 36).

Variables clínicas y parámetros laboratoriales	Intervención con romero		Tamaño del efecto	p-valor
	Antes Media ± DE	Después Media ± DE
Circunferencia Abdominal (cm)	98,08 ± 16,80	97,38 ± 15,38	0,091^a^	0,637^**^
Presión arterial sistólica (mmHg)	117,03 ± 11,10	113,28 ± 13,39	0,342^b^	0,048^*^
Presión arterial diastólica (mmHg)	78,31 ± 7,68	74,36 ± 9,10	0,423^b^	0,016^*^
Colesterol total (mg/dL)^c^	200,62 ± 29,87	185,25 ± 25,40	0,649^a^	<0,001**
Colesterol de Lipoproteína de alta densidad (mg/dl)	71,69 ± 24,11	57,41 ± 19,38	1,03^b^	<0,001^*^
Hemoglobina glicosilada (%)	5,43 ± 0,42	5,42 ± 0,33	0,054b^b^	0,746^*^

Notas: *Test T para muestras pareadas;

**Test de Wilcoxon;

^a^ Correlación biserial de órdenes;

^b^ D de Cohen;

^c^ n = 35.

Se identificó una reducción significativa en la media de la presión arterial sistólica y diastólica, con un tamaño del efecto entre pequeño y mediano. Cabe destacar que la PAS se mantuvo sin cambios en el 16,7% (n = 6), se redujo en el 50,0% (n = 18) y aumentó en el 33,3% (n = 12). La presión diastólica se mantuvo constante en el 25,0% (n = 9), disminuyó en el 55,6% (n = 20) y aumentó en el 19,4% (n = 7) de los profesionales.

Los parámetros de CT tuvieron una disminución significativa de gran efecto; entre los profesionales de enfermería, el 74,3% (n = 26) presentó una reducción tras el uso de romero y el 25,7% (n = 9) de ellos, un aumento. Los niveles de HDL-c disminuyeron significativamente, con un efecto grande. Hubo una reducción en la dosis sérica de HDL-c en el 86.1% (n = 31) de los profesionales, un aumento en el 11.1% (n = 4) y el 2.8% (n = 1) mantuvo los valores constantes.

En el análisis del ERGF, se identificó una mediana global de 3,5 puntos (IIQ = 6,00); 7,5 (IIQ = 11,5) para los hombres y 2 (IIQ = 7,0) para las mujeres, con diferencia entre los sexos (U = 34,50, p = 0,018). Para el análisis del efecto del romero sobre el ERGF, se consideraron los factores sexo y edad (Tabla 4). Se observó que los profesionales de sexo biológico masculino tienen un mayor riesgo de eventos cardiovasculares en 10 años (p < 0,001), al igual que aquellos con edad > 40 años (p < 0,001), con un efecto de gran magnitud.

**Tabla 4:** Eficacia del romero sobre la puntuación del índice de riesgo global de Framingham, comparación entre los periodos de intervención, según el sexo y la edad – Ijuí, RS, Brasil, 2022 (n = 35).

	Período	Sexo biológico		p-valor^*^ efecto del sexo (n^2^G)	p-valor^*^ interacción intervención-sexo (n^2^G)	Edad		p-valor^*^ efecto de edad (n^2^G)	p-valor^*^ interacción intervención-edad (n^2^G)	p-valor^*^ efecto de la intervención (n^2^G)	p-valor interacción intervención-sexo-edad (n^2^G)
		Masculino Media ± DE	Femenino Media ± DE			≤ 40 anos Media ± DE	> 40 anos Media ± DE				
**ERGF**	Antes	9,16 ± 6,85	2,31 ± 4,00	**<0,001** (0,382)	0,970 (0,00)	0,77 ± 2,99	8,07 ± 4,94	**<0,001** (0,474)	0,063 (0,015)	**0,047**(0,018)	0,440 (0,003)
	Después	8,17 ± 5,19	1,45 ± 3,65			0,41 ± 3,06	6,31 ± 4,57				

Nota: ERGF: puntuación global de riesgo de Framingham; DE: desviación estándar;

^*^ANOVA de efecto mixto y tamaño del efecto: Eta cuadrado generalizado (n^2^G).

La intervención con romero redujo significativamente la puntuación del ERGF (p = 0,047); sin embargo, el tamaño del efecto fue pequeño (n2G = 0,018). Cabe destacar que el 28,6% (n = 10) de los profesionales mantuvieron el ERGF sin cambios entre los periodos de observación, el 51,4% (n = 18) presentaron una disminución y el 20,0% (n = 2) un aumento.

## DISCUSIÓN

El principal resultado de este estudio es que la ingesta de cápsulas de extracto seco de *Rosmarinus officinalis L*. (1 g/día) puede contribuir a reducir el riesgo de eventos cardiovasculares en los profesionales de enfermería de los servicios de urgencias, principalmente porque favorece la disminución de la presión arterial y del colesterol total y tiene pocos efectos adversos, pero los resultados son preliminares y deben interpretarse con cautela. Se reconoce que las ECV son la principal causa de morbilidad y mortalidad en Brasil y en el mundo, y entre sus factores de riesgo destacan la dislipidemia y la hipertensión^(19)^; los fitoterápicos eficaces en la reducción de estos factores pueden tener un impacto positivo.

Los resultados contribuyen a la consolidación de la ciencia de la enfermería; amplían las evidencias sobre el uso del romero en el tratamiento de los factores de riesgo de aterosclerosis y enfermedades cardiovasculares, poco explorados hasta el momento; contribuyen al desarrollo científico basado en la diversidad cultural; validan el conocimiento popular sobre los beneficios del romero; y estimulan el protagonismo de la enfermería en la investigación con fitoterápicos.

Los hallazgos de esta investigación se basaron en una muestra en la que las características sociolaborales en cuanto a edad y sexo se asemejan al perfil de la enfermería en el mundo, que es predominantemente femenina y relativamente joven^(20)^. Su nivel educativo, con prevalencia de educación superior o superior, aunque la mayoría de los profesionales ocupaban puestos de nivel medio, refleja los cambios en el mercado laboral de la enfermería, que exige profesionalización, especialización y estimula la competitividad^(21)^.

El ingreso familiar mensual promedio de aproximadamente 1618,68 dólares estadounidenses (± 677,15 dólares estadounidenses) o 5838,00 reales brasileños permitió clasificar a la enfermería en la clase media^(22)^, lo que parece ser un buen indicador; sin embargo, se observó que la mayoría de los profesionales cumplían una jornada laboral superior a 44 horas semanales y trabajaban en turnos rotativos, con riesgo para su salud. En este sentido, el estudio identificó una asociación entre el trabajo diurno con regreso al servicio en menos de 11 horas de forma rutinaria y el riesgo de IAM^(23)^.

Se observó que más del 50% de los profesionales presentaban sobrepeso u obesidad, aumento del perímetro abdominal, alteraciones en los niveles de colesterol total y casi el 30% presentaba alteraciones en la presión arterial sistémica. Estos factores de riesgo pueden estar relacionados con la organización del trabajo de enfermería. La investigación refuerza esta afirmación al indicar una asociación entre el trabajo por turnos y el aumento de la presión arterial, el colesterol total y el IMC^(7)^. Del mismo modo, la desregulación de la secreción de cortisol y el estrés crónico, comunes entre estos profesionales, están relacionados con los cambios epigenéticos implicados en la dislipidemia, la resistencia a la insulina, la adiposidad, la respuesta inmunológica deteriorada y el aumento de la inflamación sistémica^(24)^.

A pesar de este contexto, los participantes presentaron un bajo riesgo de eventos cardiovasculares en 10 años. Se identificó una relación entre la estimación del riesgo cardiovascular y la edad y el sexo, mayor en los hombres y en los mayores de 40 años. Este hallazgo contribuye a las pruebas actuales de que la incidencia de enfermedades cardiovasculares aumenta con el envejecimiento de la población y que la tasa mundial de mortalidad por ECV entre los hombres es mayor en comparación con las mujeres^(1)^.

La edad cronológica es un factor de riesgo, pero existe una interacción y sinergia entre la edad cronológica y los factores de riesgo modificables en el desarrollo de ECV, lo que refuerza la importancia de las medidas preventivas y promotoras del envejecimiento saludable. En relación con el sexo, se entiende que, aunque la prevalencia de las ECV es menor en las mujeres que en los hombres, después de un evento cardiovascular agudo, las mujeres presentan una mayor tasa de mortalidad y un peor pronóstico. También destaca la influencia de la expresión genética de los cromosomas sexuales y las diferencias en las hormonas sexuales en la fisiopatología y la incidencia de eventos cardiovasculares^(25)^.

En cuanto a la eficacia del extracto seco de romero sobre los factores de riesgo de ECV, al comparar el periodo anterior y posterior a la intervención, se observó una reducción significativa de la presión arterial sistólica y diastólica de los profesionales de enfermería.

Los compuestos del romero, como el ácido rosmarínico, que actúan en la disminución de la angiotensina II y de la enzima convertidora de angiotensina relacionadas con el aumento de la presión arterial^(26)^, pueden haber contribuido a la reducción de la presión arterial sistólica y diastólica de los participantes.

Teniendo en cuenta la elevada prevalencia de hipertensión arterial en la población y la relación causal con la cardiopatía isquémica y la mortalidad por accidente cerebrovascular, que repercuten en años de vida perdidos por discapacidad^(19)^, el potencial hipotensor del romero puede contribuir a la reducción de estos resultados. Sin embargo, el efecto observado en la muestra analizada fue de pequeño a mediano, lo que indica una importancia clínica y práctica limitada.

En la presente investigación, no se identificó una diferencia estadísticamente significativa entre los periodos de intervención sobre la circunferencia abdominal, aunque hubo una reducción en sus valores medios, lo que puede estar relacionado con la dosis utilizada y/o el tiempo de intervención. Un estudio con mujeres obesas que consistió en la ingesta de cápsulas con aceite de romero en una dosis de 380 mg/día durante seis semanas, junto con una dieta, mostró una reducción de la relación cintura-cadera^(27)^, al igual que se observó en personas con DM que tomaron té de romero en una dosis de 2 g/día durante 12 semanas^(13)^. Teniendo en cuenta que la relación cintura-cadera está asociada con el sobrepeso, el síndrome metabólico y la DM, la reducción de esta medida, vinculada al uso del romero, puede repercutir en la reducción del riesgo cardiovascular.

Los niveles de colesterol total también fueron menores después de la intervención, con un efecto de gran magnitud, lo que sugiere su importancia clínica. Se observó un hallazgo similar en un estudio que evaluó el efecto del romero en polvo en dosis de 5 y 10 g/día durante 8 semanas sobre el perfil lipídico de personas sanas^(15)^. El mecanismo de acción del romero, implicado en la disminución del colesterol y los triglicéridos, está relacionado con su capacidad antiinflamatoria y antioxidante, que interfiere en el metabolismo lipídico, inhibe la lipólisis y la maduración de los adipocitos, reduce la síntesis de colesterol y ácidos grasos en el hígado y el tejido adiposo y aumenta su depuración^(28)^.

A partir del mecanismo de acción del romero, se puede inferir que los resultados de la presente investigación refuerzan las evidencias sobre el potencial de la planta para la salud vascular, ya que la participación del colesterol en los procesos ateroscleróticos está relacionada con la capacidad individual de absorción de este lípido, con el tipo de absorción que contribuye a la composición y calidad de las partículas de LDL-c, y con su eliminación ineficaz^(29)^.

En cuanto al HDL-c, en la comparación entre los periodos de intervención, se observó una reducción significativa del parámetro, con un efecto importante. Las investigaciones han sugerido que, antes de la menopausia, los niveles elevados de HDL-c son cardioprotectores para las mujeres, pero no para aquellas que se encuentran en transición hacia la menopausia. La reducción de los niveles de estradiol influye en factores que afectan a la calidad de la partícula de HDL-c, su composición y función, convirtiéndola en un factor proinflamatorio y proaterogénico, independientemente de la edad, la raza/etnia, la educación, el IMC, la PAS, el LDL-c, el uso de medicamentos para las ECV y la proteína C reactiva^(30)^.

Una investigación realizada con adultos chinos evidenció una asociación en forma de U entre el HDL-c y la mortalidad por todas las causas, por ECV y por cáncer. Se demostró que una de cada treinta muertes por todas las causas puede atribuirse a niveles elevados y bajos de HDL-c y que los valores entre 50 y 79 mg/dL representan un menor riesgo de mortalidad por todas las causas y por ECV.

Los niveles altos y bajos de HDL-c se asociaron con un aumento de la mortalidad por cardiopatía isquémica y enfermedad isquémica cerebrovascular. Los autores también señalan el potencial inflamatorio y aterogénico de la molécula cuando presenta alteraciones en la composición proteica y se vuelve disfuncional^(31)^. Ante esto, se sugiere un efecto beneficioso del romero sobre este resultado, ya que el promedio de los valores de HDL-c de los participantes se mantuvo dentro del rango normal. Sin embargo, el efecto del romero sobre esta molécula debe investigarse a largo plazo, considerando que la reducción progresiva de los niveles de HDL-c puede ser perjudicial para la salud cardiovascular y suscitar dudas sobre la seguridad de la planta.

No se observaron cambios significativos en la hemoglobina glicosilada entre los periodos de intervención, en consonancia con el estudio realizado con personas sanas y con diabetes^(14)^, en el que el uso de 3 g/día de polvo de *Rosmarinus officinalis L.* encapsulado durante 8 semanas por personas sanas no presentó diferencias significativas con la intervención. Por otro lado, estudio sugiere que el romero y sus compuestos fenólicos actúan mejorando la resistencia a la insulina, suprimiendo la gluconeogénesis, estimulando la captación de glucosa y tienen un efecto antiinflamatorio y antioxidante^(32)^, lo que apunta a la necesidad de investigaciones con un mayor tiempo de intervención o con dosis diferentes a las investigadas para aclarar la eficacia del romero sobre este parámetro.

Con la intervención, se identificó una reducción significativa en la puntuación del ERGF, pero con una importancia clínico-práctica limitada. Cabe destacar que el 51,4% de los profesionales presentaron una disminución en la puntuación, lo que puede estar asociado a la reducción de los parámetros de presión arterial y colesterol total identificados en los participantes, considerando que el algoritmo toma en cuenta dichas variables para estimar el riesgo^(2)^.

En el mundo, más de 500 millones de personas se ven afectadas por enfermedades cardiovasculares, principalmente cardiopatías isquémicas y accidentes cerebrovasculares, y los avances médicos en este ámbito se concentran en países de altos ingresos^(1)^. Las investigaciones destinadas a proporcionar pruebas sobre una terapia complementaria o adyuvante en el tratamiento de los factores de riesgo de estas enfermedades, de fácil acceso para la población, en un país subdesarrollado, son una innovación que contribuye a la reducción de la mortalidad prematura por enfermedades crónicas no transmisibles, objetivo de los Objetivos de Desarrollo Sostenible.

En relación con la intervención con romero, el análisis de su eficacia sobre los resultados de salud de los profesionales de enfermería, con miras a la seguridad de los participantes, se realizó junto con el seguimiento y la identificación de eventos adversos. Posiblemente, eventos como somnolencia, mejora de la piel del rostro, taquicardia, ansiedad, irritabilidad, llanto frecuente, cefalea y dolor de estómago/acidez estomacal estuvieron relacionados con la intervención, a diferencia de lo observado en un estudio con personas privadas de libertad^(33)^, en el que, con una intervención similar, no se identificaron eventos adversos. Estos hallazgos refuerzan la necesidad de más investigaciones que evalúen su seguridad y eficacia en diferentes resultados. Señalan la importancia de las acciones de promoción del uso racional y la educación para el uso correcto de la preparación botánica, el respeto a la dosis, la vía de administración y el período de uso, y la desmitificación de la inocuidad.

Diferentes preparaciones botánicas, como el extracto seco y el aceite esencial, pueden provocar efectos diferentes en el organismo, al igual que la concentración de determinados compuestos químicos en estas sustancias. Un ejemplo de ello fue el resultado presentado por una investigación con romero dirigida a personas con diagnóstico de hipotensión primaria, en la que 1 ml de aceite esencial en un terrón de azúcar cada 8 horas durante 44 semanas mostró una tendencia al aumento de la presión arterial^(34)^, a diferencia del efecto hipotensor encontrado en este estudio con el extracto seco con un contenido activo de flavonoides superior a 0,03.

Es importante destacar que el aceite esencial es un producto aromático, volátil, un metabolito secundario utilizado por las plantas como protección, cuya eficacia depende de la concentración y la interacción sinérgica de sus componentes químicos, influenciada por los procesos de extracción, el entorno de desarrollo de la planta y otros factores^(35)^. El extracto seco, por su parte, es una preparación sólida obtenida a partir de drogas vegetales, resultado de la evaporación del solvente utilizado en el proceso de extracción y de la eliminación de materiales indeseables, y que cuenta con especificaciones de componentes químicos (a efectos de control de calidad)^(16)^.

La realización de esta investigación presentó algunas limitaciones, entre ellas: el pequeño tamaño de la muestra debido a las pérdidas de seguimiento, lo que puede introducir un sesgo de fricción; el ERGF, que tiene baja sensibilidad para predecir eventos a corto plazo; la falta de un seguimiento sistemático de variables conductuales relevantes, como la alimentación, la práctica de actividad física, el uso concomitante de medicamentos y los niveles de estrés o la calidad del sueño, con un posible sesgo de confusión; la ausencia de un grupo de control; y la evaluación de la eficacia del romero sobre la hemoglobina glicosilada con una intervención que duró menos de tres meses, lo que puede haber influido en los resultados. Además, el pequeño tamaño del efecto observado sobre el ERGF y la presión arterial indica un efecto clínico limitado para estos resultados. A pesar de ello, los resultados aportaron pruebas con potencial para producir un impacto social, económico y en los indicadores de morbimortalidad.

Dadas las limitaciones y al tratarse de un estudio preliminar, la interpretación de los resultados debe hacerse con cautela. Se necesitan nuevos ensayos con una muestra más amplia, un grupo de control y un seguimiento a largo plazo para confirmar la eficacia y la seguridad del uso del romero como estrategia para reducir el riesgo cardiovascular.

## CONCLUSIÓN

La administración oral de cápsulas de extracto seco de *Rosmarinus officinalis L*. (1 g/día) se mostró eficaz en la reducción de la estimación del riesgo cardiovascular a 10 años de los profesionales de enfermería de los servicios de urgencias, lo que evidencia su potencial para el tratamiento y control de los factores de riesgo cardiovascular. Se identificó una reducción de la presión arterial sistólica y diastólica, de los niveles de colesterol total y de colesterol de lipoproteínas de alta densidad, con efecto ateroprotector y pocos eventos adversos. Sin embargo, el tiempo de intervención y/o la dosis utilizada pueden no haber sido suficientes para mejorar el perfil glucídico y producir un efecto clínico importante sobre el ERGF y la presión arterial. Las limitaciones del estudio y la escasa exploración del tema requieren más investigaciones sobre el tema dirigidas a confirmar la eficacia y la seguridad del uso del romero para la salud cardiovascular.

## Data Availability

El conjunto de datos que respaldan las conclusiones de este estudio está disponible previa solicitud al autor correspondiente.
